# Medial prefrontal cortical PPM1F alters depression‐related behaviors by modifying p300 activity via the AMPK signaling pathway

**DOI:** 10.1111/cns.14293

**Published:** 2023-06-12

**Authors:** Jing Liu, Fantao Meng, Wentao Wang, Min Wu, Yu Zhang, Minghu Cui, Changyun Qiu, Fengai Hu, Di Zhao, Dan Wang, Cuilan Liu, Dunjiang Liu, Zhicheng Xu, Yameng Wang, Wei Li, Chen Li

**Affiliations:** ^1^ Department of Rehabilitation Medicine Binzhou Medical University Hospital Binzhou Shandong China; ^2^ Medical Research Center Binzhou Medical University Hospital Binzhou Shandong China; ^3^ Institute for Metabolic & Neuropsychiatric Disorders Binzhou Medical University Hospital Binzhou Shandong China; ^4^ Department of Psychology Binzhou Medical University Hospital Binzhou Shandong China; ^5^ Department of Neurosurgery Binzhou Medical University Hospital Binzhou Shandong China; ^6^ College of Nursing Binzhou Medical University Binzhou Shandong China

**Keywords:** depression‐related behaviors, mPFC, neuroinflammation, neuronal excitability, PPM1F‐AMPK‐p300 axis

## Abstract

**Aims:**

Protein phosphatase Mg2+/Mn2+‐dependent 1F (PPM1F) is a serine/threonine phosphatase, and its dysfunction in depression in the hippocampal dentate gyrus has been previously identified. Nevertheless, its role in depression of another critical emotion‐controlling brain region, the medial prefrontal cortex (mPFC), remains unclear. We explored the functional relevance of PPM1F in the pathogenesis of depression.

**Methods:**

The gene expression levels and colocalization of PPM1F in the mPFC of depressed mice were measured by real‐time PCR, western blot and immunohistochemistry. An adeno‐associated virus strategy was applied to determine the impact of knockdown or overexpression of PPM1F in the excitatory neurons on depression‐related behaviors under basal and stress conditions in both male and female mice. The neuronal excitability, expression of p300 and AMPK phosphorylation levels in the mPFC after knockdown of PPM1F were measured by electrophysiological recordings, real‐time PCR and western blot. The depression‐related behavior induced by PPM1F knockdown after AMPKα2 knockout or the antidepressant activity of PPM1F overexpression after inhibiting acetylation activity of p300 was evaluated.

**Results:**

Our results indicate that the expression levels of PPM1F were largely decreased in the mPFC of mice exposed to chronic unpredictable stress (CUS). Behavioral alterations relevant to depression emerged with short hairpin RNA (shRNA)‐mediated genetic knockdown of PPM1F in the mPFC, while overexpression of PPM1F produced antidepressant activity and ameliorated behavioral responses to stress in CUS‐exposed mice. Molecularly, PPM1F knockdown decreased the excitability of pyramidal neurons in the mPFC, and restoring this low excitability decreased the depression‐related behaviors induced by PPM1F knockdown. PPM1F knockdown reduced the expression of CREB‐binding protein (CBP)/E1A‐associated protein (p300), a histone acetyltransferase (HAT), and induced hyperphosphorylation of AMPK, resulting in microglial activation and upregulation of proinflammatory cytokines. Conditional knockout of AMPK revealed an antidepressant phenotype, which can also block depression‐related behaviors induced by PPM1F knockdown. Furthermore, inhibiting the acetylase activity of p300 abolished the beneficial effects of PPM1F elevation on CUS‐induced depressive behaviors.

**Conclusion:**

Our findings demonstrate that PPM1F in the mPFC modulates depression‐related behavioral responses by regulating the function of p300 via the AMPK signaling pathway.

## Graphical abstract

Chronic unpredictable stress decreased the expression levels of PPM1F in the mPFC, which activated the AMPK signaling pathway, downregulated the expression levels and acetylase activity of p300, and resulted in the formation of depression.

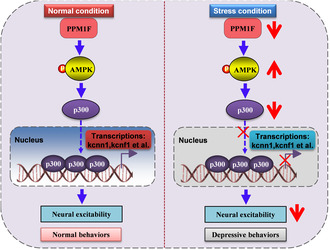

## 1 INTRODUCTION

Depression is a common and debilitating psychiatric disorder, with core features of distinct and persistent loss of interest and low mood. Depression affects over 350 million people worldwide and can result in serious dysfunction in patients.^1^, ^2^ Currently, although pharmacological therapies for major depressive disorder are available, the limitations of medical treatments for depression often show a high‐rate ineffectiveness, and are concomitant with intolerable side effects.^3^, ^4^ Thus, a deeper exploration of the pathogenic mechanisms of depression is urgently required to provide insights for the development of novel therapeutic drugs.

The medial prefrontal cortex (mPFC) is a cortical region with different cell types and projections that innervate numerous brain areas, and is recognized to play an essential role in intellectual emotional control, including in relation to anxiety and depression.^5^ Volumetric reductions in the mPFC are among the most well‐documented neural abnormalities in patients with major depressive disorder.^6^ Further, protein phosphatase Mg2+/Mn2+‐dependent 1F (PPM1F) is a serine/threonine phosphatase from the protein phosphatase 2C family,^7^, ^8^ and its functional activity has been linked to apoptosis regulation, proliferation and metastasis of cancer cells.^9^, ^10^ Previous research has identified six PPM1F single‐nucleotide polymorphisms that affect the association between posttraumatic stress disorder symptom severity and cortical thickness of the bilateral superior frontal and orbitofrontal regions, suggesting that variants of this gene may be relevant to the neural integrity of the prefrontal cortex (PFC).^11^ Our previous research found that dysfunctional elevation of PPM1F expression in the hippocampus is a crucial factor affecting depression and anxiety, and bidirectional modulation of PPM1F expression in the dentate gyrus can produce diverse phenotypes associated with depression and anxiety.^12^, ^13^ In addition, an association between dysfunctional levels of PPM1F in the mPFC and depression and anxiety has been revealed in both animals and humans.^14^, ^15^ However, it remains unclear whether PPM1F in the mPFC is involved in the pathogenesis of major depression.

p300 is a histone acetyltransferase (HAT) that acetylates histones and a growing list of transcription‐related proteins to promote transcription activity by loosening chromatin, enhancing their DNA‐binding activity, and facilitating protein–DNA recognition and protein–protein interactions.^16^, ^17^ Further, p300 is reportedly linked to the pathogenesis of several cancer modalities and is critical for both object recognition and contextual fear memory, and neurodegenerative disorders.^16^, ^18^, ^19^ Marek et al. reported that infusing a small‐molecule p300‐specific inhibitor (C646) into the infralimbic cortex of the PFC, in which p300 is highly expressed within pyramidal neurons, enhances the consolidation of fear extinction memory by accelerating long‐term potentiation under weak extinction training.^20^ However, considering the important role of p300, limited research has been conducted on its function within the context of psychiatric disorders, especially for depression. Therefore, an extensive examination of the neural and cellular mechanisms of p300 is needed.

In this study, we first established a depression‐related animal model induced by chronic unpredictable stress (CUS) and analyzed the gene expression levels of PPM1F in the mPFC in relation to depressive behaviors. We then used an adeno‐associated virus strategy to determine the impact of knockdown or overexpression of PPM1F in the excitatory neurons on depression‐related behaviors under basal and stress conditions in both male and female mice. In addition, we tested whether knockdown of PPM1F regulates the neuronal excitability of pyramidal neurons in the mPFC, and identified the causal relationship between neuronal excitability and depression‐related behaviors. We also examined the expression levels of p300 after PPM1F knockdown, accompanied by abnormal AMPK phosphorylation levels, and tested the depression‐related behaviors induced by PPM1F knockdown after AMPKα2 knockout. Finally, we investigated whether the acetylation activity of p300 was required for the antidepressant activity of PPM1F overexpression.

## 2 MATERIALS AND METHODS

### 2.1 Animals

Wild‐type (WT) C57BL/6J mice (Stock No. 000664) and AMPKα2^flox/+^ mice (Stock No. 014142),^21^ which possess loxP sites flanking the region encoding exon 2 of AMP‐activated protein kinase (AMPK), were purchased from Jackson Laboratory (Bar Harbor, ME, USA). All the mice were backcrossed and maintained to a C57BL/6J background. Heterozygous flox/+ transgenic mice were crossed to produce homozygous offsprings. For genotyping, the following PCR primers were used: AMPKα2 WT and flox: forward‐5′‐GCAGGC GAATTTCTGAGTTC‐3′, reverse‐5′‐TCCCCTTGAACAAGCATACC‐3′. The animals were housed in groups of 3–5 per cage under a 12 h light/dark cycle (lights on at 7:00 a.m.) with water ad libitum and standard food pellets. Both male and female mice (8–12 weeks old) were used. This study was approved by the Institutional Animal Care and Use Committee of Binzhou Medical University Hospital^22^ and conducted in accordance with the U.K. Animals (Scientific Procedures) Act, 1986 and associated guidelines, EU Directive 2010/63/EU for animal experiments.

### 2.2 Drugs

C646, a specific inhibitor of histone acetyltransferase p300 (p300 HAT; Sigma‐Aldrich), was dissolved in dimethyl sulfoxide and injected at a dose of 3 μg/μL.^23^

### 2.3 Stereotaxic surgery, microinjection, and cannulation

The coding region of mouse PPM1F (NM_176833) with calcium/calmodulin‐dependent protein kinase II (CaMKII) promoter or the short hairpin RNA oligonucleotides GGATGAGAAAGCACGAATTGA and GCATACCAATGCTTCTCACCA targeted PPM1F with an independent U6 promoter were packaged into an adeno‐associated virus (AAV2/9) with titers >1 × 10^12^ vg/mL (Hanbio, shanghai, China). The oligonucleotide TTCTCCGAACGTGTCACGT was used as a nonspecific control (NC). The efficiency and specificity of overexpression or knockdown of PPM1F were confirmed using quantitative real‐time PCR.^12^

All surgeries were conducted using a stereotaxic apparatus (Kopf Instrument) under deep anesthesia conditions using isoflurane. AAV2/9‐CaMKII‐Cre‐GFP, AAV2/9‐CaMKII‐GFP, AAV2/9‐CaMKII‐Cre‐mCherry, AAV2/9‐CaMKII‐mCherry, AAV2/9‐hSyn‐hM3D(Gq)‐mCherry (AAV‐hM3‐mCherry), AAV2/9‐CaMKII‐PPM1F‐GFP (AAV‐PPM1F‐GFP), AAV2/9‐U6‐shRNA‐GFP (AAV‐shRNA‐GFP), and AAV2/9‐U6‐NC‐GFP (AAV‐NC‐GFP) were infused bilaterally into mPFC at the coordinates corresponding to: AP = +1.78 mm, ML = ±0.4 mm, DV = −2.60 mm from the bregma of adult WT or AMPKα2^flox/flox^ mice. In addition, 0.5 μL adeno‐associated viral (AAV) vectors (per side) were injected at a controlled rate of 100 nL/min using a mineral oil‐filled glass micropipette with a UMP3 microsyringe pump (World Precision Instruments). Behavioral tests were conducted 3 weeks after viral injection. For all viral infections, animals with incorrect injection sites were excluded from data analysis.

For intra‐mPFC microinjection of the C646, bilateral guide cannula (23‐gauge; RWD Life Science CO., Ltd.) was implanted 1 mm above the mPFC (coordinates: AP = +1.78 mm, ML = ± 0.4 mm, DV = −1.6 mm from the bregma) of adult male C57BL/6J mice (8‐week old) as previously described.^24^ Microinjections were performed on freely moving mice in their home cage. A bilateral injection cannula (33‐gauge) connected to a 5‐μL syringe was inserted into the guide cannula. C646 or vehicle was infused into the mPFC in a volume of 0.5 μL over 5 min using an infusion pump (KD Scientific Inc.). The injector tips were held in place for another more 5 min after the end of injection to avoid backflow. The mice with loosed or missed canulations in each group were not included in the statistical analysis.

### 2.4 Immunofluorescence histochemistry analysis

Mice were deeply anesthetized and transcardially perfused with ice‐cold PBS followed by 4% paraformaldehyde. The intact brains were fixed in 4% paraformaldehyde for 24 h at 4°C. After being washed with PBS, the brains were stored in a 30% sucrose solution for 48 h at 4°C. Frozen brains were sectioned at 40 μm thickness. After three washes with PBS, slices were blocked for 1 h in an immunoblocking buffer (1% bovine serum albumin, 0.3% goat serum, 3% Triton X‐100 in PBS) at room temperature, and then incubated with PPM1F (1:400, PA5–15571, Invitrogen) and anti‐CaMKII antibody (1:500, ab134041, Abcam) in blocking solution overnight at 4°C. After being washed three times with PBS, the slices were incubated with secondary antibodies, Alexa Fluor®488 goat anti‐rabbit IgG antibodies (1:400, A21202, Invitrogen) or Alexa Fluor® 546 goat anti‐rabbit IgG antibodies (1:400, A11035, Invitrogen) at room temperature for 4 h. Slides were sealed with anti‐fluorescence quencher and visualized using an Olympus FV1000 confocal microscope (Olympus). Florence positive cells in 2–5 sections from each brain from approximately 1.54 to 1.98 mm for mPFC relative to bregma were calculated manually by researchers who were blinded to the experimental designs.^25^

### 2.5 Real‐time PCR analysis

Brain samples were dissected to collect the mPFC following guidelines provided in *The Mouse Brain Atlas in Stereotaxic Coordinates* (Keith Franklin, George Paxinos, 2008, Academic Press)^24^ and quickly frozen in liquid nitrogen, and stored at −80°C. Total RNA was extracted using Tissue RNA kit (Omega), following manufacturer's instructions. The cDNA was generated using 5 × HiScript II QRT SuperMix (Vazyme, Nanjing, China) and incubated at 25°C for 10 min, 50°C for 30 min, and 85°C for 5 min. The resulting cDNA was processed for real‐time PCR quantification using the StepOnePlus real‐time PCR system (Applied Biosystems). The sequences of primers used for real‐time PCR are listed in Table S1. The 2^−ΔΔCT^ analysis was used for relative quantification.^12^

### 2.6 Western blot analysis

Total proteins were extracted as previously described.^23^, ^26^, ^27^ The tissues were briefly homogenized in radioimmunoprecipitation assay (RIPA) lysis buffer (Biotime) supplemented with 1% phenylmethylsulfonyl fluoride (Sangon Biotech) and 1× PhosSTOP phosphatase inhibitor cocktail (Roche Applied Science). Protein concentration was measured using a butyleyanoacrylate (BCA) kit according to the manufacturer's instructions (Thermo Scientific). The proteins were separated using SDS‐polyacrylamide gel electrophoresis (SDS‐PAGE) and then transferred onto a polyvinylidene fluoride (PVDF) membrane (Millipore). The membranes were blocked in 5% bovine albumin (BSA) with a TBST buffer (20 mM Tris–HCl, pH 7.4, 150 mM NaCl, 0.1% Tween 20), followed by incubation with primary antibodies diluted in TBST containing 2.5% BSA at 4°C overnight: anti‐PPM1F (1:500, PA5‐15571, Invitrogen), anti‐p‐AMPK antibody (1:1000, #2535, Cell Signaling Technology), anti‐AMPK antibody (1:1000, ab32047, Abcam), and anti‐β‐actin antibody (1:1000, 4970, Cell Signaling Technology). Then, the membranes were washed with 1× TBST and incubated with IRDye 680LT donkey anti‐rabbit IgG secondary antibodies (1:5000, 926–68,023, Li‐COR Biosciences). Fluorescence was visualized and analyzed using an Odyssey infrared imaging system (Li‐COR Biosciences).

### 2.7 Electrophysiological recordings

Electrophysiological recordings were conducted as previously described.^24^ First, the brains from the anesthetized mice with isoflurane were quickly transferred to an ice‐cold solution (254 mM sucrose, 3 mM KCl, 2 mM MgCl_2_, 2 mM CaCl_2_, 1.25 mM NaH_2_PO_4_, 10 mM D‐glucose, and 24 mM NaHCO_3_). Coronal brain slices (300 μm) containing the mPFC were cut using a Leica VT1200 vibratome (Leica Microsystems) and left to recover at 30°C for at least 1 h in artificial cerebrospinal fluid extracellular solution (124 mM NaCl, 2 mM KCl, 2 mM MgSO_4_, 1.25 mM NaH_2_PO_4_, 2 mM CaCl_2_, 26 mM NaHCO_3_, 10 mM D‐dextrose, and 0.4 mM vitamin C) under oxygen incubation (95% O_2_/5% CO_2_). Patch electrodes with tip resistances between 4–7 MΩ were filled with a potassium gluconate‐based internal solution (120 mM potassium gluconate, 20 mM KCl, 2 mM MgCl_2_, 10 mM HEPES, 2 mM ATP, 0.25 mM GTP, and 0.1 mM EGTA adjusted to 7.4, and osmolarity of 295 mOsm). The neurons, which were identified using infrared microscopy and a charge‐coupled device camera, were clamped in whole‐cell mode and recorded with a multiclamp 700 B amplifier (Molecular Devices). Data collection was performed using a hardware (clampfit) filter of 3 kHz. Current‐clamp recordings were made from a −80 mV holding current and incremental stepwise current injections (10 pA) for a duration of 500 ms to record the firing properties. The action potentials were counted for each current injection for 500 ms at steady firing rates. The passive membrane properties of the cells were analyzed at resting membrane potential. The input resistance was measured using a hyperpolarizing current injection of −20 pA from a −80 mV holding current. The fast after hyperpolarization (fAHP) size was tested as the difference between the spike threshold and voltage minimum after the action potential peak.

### 2.8 Chronic unpredictable stress and behavioral procedures

The CUS procedure was carried out as described previously with minor modification^28^, ^29^ to maximize the unpredictability and mildness of the stress intensity. Briefly, mice were subjected to two different stressors at different times of every day for 14 consecutive days, which included restraint (2‐h), tail pinch (15‐min), constant light (24‐h), wet bedding (24‐h) with 45° cage tilt, inescapable foot shocks (10‐min, 0.3 mA, 2 s duration, the interval is 16 s), elevated platform (30‐min), and social isolation.^30^ Control mice were group housed and briefly handled daily in the housing room. An abbreviated (7 days) subchronic unpredictable stress (SCUS) was conducted to assess stress susceptibility.^28^

All behavioral tests were performed during the late light phase of the lighting cycle, except for the sucrose preference test, which was conducted in the initial 2 h of the dark phase. Prior to each test, all mice were habituated to the testing room for 4 h. To avoid possible subjective effects, the behavioral performance of each mouse in each test was recorded using a double‐blind procedure. To avoid possible carryover effects, the adjacent behavioral tests were spaced apart by 2–3 days. The procedures used for the behavioral tests are presented sequentially below.

#### 2.8.1 Female urine sniffing test

We used a female urine sniffing test (FUST) to examine the sex‐related reward‐seeking behavior of male mice based on their interest in pheromonal odors from estrus female urine.^31^ As previously described,^12^, ^26^ estrus female urine was collected by monitoring the estrus cycle of female mice using microscopic examination of vaginal smears. Before testing, male mice were habituated to a sterile cotton‐tipped applicator inserted into their home cage for 1 h before being transferred to a testing room with constant dim light (approximately 3 lux). The test was conducted in three stages: (1) exposure to a cotton‐tipped applicator dipped in sterile water for 3 min, (2) a 45‐min interval, and (3) exposure to a cotton‐tipped applicator dipped in fresh urine (80 μL) of estrus female mice for 3 min. The sniffing times for sterile water and female urine were recorded respectively.

#### 2.8.2 Sucrose preference test (SPT)

As described previously,^28^ 1 week before testing, mice were habituated to drinking water from two tubes (50 mL) with stoppers fitted with ball‐point sipper tubes. Four hours before testing, mice were transferred to the testing room from the group to individual housing without water. During testing, a free choice between a tube of either water or sucrose solution (1%) was presented to mice for 2 h, and the water and sucrose solution consumption were recorded. The preference for sucrose solution was calculated as the ratio of the consumption of sucrose solution to the total consumption of water and sucrose solution.

#### 2.8.3 Forced swim test (FST)

The apparatus of the forced swim test (FST) was a clear Plexiglas cylinder (10 cm in diameter × 25 cm in height) filled with warm water (24°C, 15 cm in depth). At the beginning of the test, the mice were placed individually into the cylinder for 6 min. The total duration of immobility was recorded during the last 4 min of the 6 min test using a video camera positioned directly above the cylinder. Immobility was defined as the absence of any body or limb movement except for those caused by respiration.

#### 2.8.4 Novelty suppressed food test (NSFT)

A novelty suppressed food test (NSFT) was conducted according to previously published protocols.^32^, ^33^ Mice were deprived of food for 24 h. Testing was performed in a plastic box (60 × 60 × 40 cm^3^) covered with bedding and illuminated with dim light. A single pellet of food on round filter paper (11 cm in diameter) was placed in the center of the box, and a mouse was placed in the corner. The latency period before eating was recorded for 10 min. The mice were then returned to their home cages. Food consumption was calculated after 5, 10, and 30 min.

#### 2.8.5 Elevated plus maze test (EPM)

According to the previous paper,^25^ the maze consisted of four arms arranged around a central platform (5 × 5 cm^2^) that with open access to any arm with white Plexiglas. Testing was conducted under bright white light. The test was initiated by lacing each mouse in the central area of the maze facing the corner between a closed arm and an open arm, and allowed to explore the elevated plus maze for 5 min. The maze was thoroughly cleaned with 20% ethanol after each trial. The time spent on the open and closed arms and the number of entries into each arm were scored. The anxiety‐related behavior was evaluated by calculating the percentage of open arm time (time spent in the open arms / total time spent in all arms) and the percentage of open arm entries (entries into the open arms / total entries into all arms).

#### 2.8.6 Open field test (OFT)

An open field test (OFT) was performed in a nontransparent square box with an open area of 60 × 60 cm^2^ and walls 40 cm in height. The entire test area was adjusted to even illumination with a house light on the ceiling. At the beginning of each session, mice were placed in the center of the open field area, and their activity was recorded for 5 min. The arena was thoroughly cleaned with 20% ethanol between tests. For the analysis, the open field arena was divided into nine equal squares using a 3 × 3 grid. The assessed parameters were time spent in the center zone and total distance traveled, quantified using Any‐maze software (Stoelting).

#### 2.8.7 Locomotor activity

The locomotor activity of the mice was assayed in SuperFlex Fusion open‐field cages (40 × 40 × 30 cm, Omnitech Electronics Inc.). A single mouse was gently placed in the corner of the cage and allowed to explore the field freely under illuminated conditions for 30 min. Mouse movements were monitored and recorded using infrared motion sensors attached to the tops of the cages. The total distance traveled was quantified using Automated Fusion Software (Omnitech Electronics Inc.).

### 2.9 Statistical analyses

All statistical analyses were performed using GraphPad Prism version 8. The normality and equal variance assumptions were tested using the Shapiro–Wilk and *F* tests. Two‐tailed unpaired *t*‐tests were used to assess the differences between the two experimental groups for normally distributed data. Two‐tailed *t*‐tests with Welch's correction were used to analyze the normally distributed data with unequal variances, whereas Mann–Whitney U tests were used for non‐normally distributed data. For the analysis of the three groups, one‐way analyses of variance (ANOVAs) followed by Sidak post hoc tests were used for normally distributed data. The Kruskal–Wallis test followed by Dunn's multiple comparisons test was used for non‐normally distributed data. For multiple groups, two‐way or two‐way repeated‐measures ANOVAs were used followed by Tukey's test. The linear relationships between two variables were analyzed by calculating Pearson's correlation coefficient, the 95% confidence bands were indicated by dotted lines. Statistical significance was set at *p* < 0.05. All data are presented as means ± standard errors (SEM). A Grubbs outlier test was performed, and samples that varied by >2 standard deviations from the mean were removed.

## 3 RESULTS

### 3.1 Dysfunctional regulation of PPM1F expression in the mPFC of depressed mice

**FIGURE 1:**
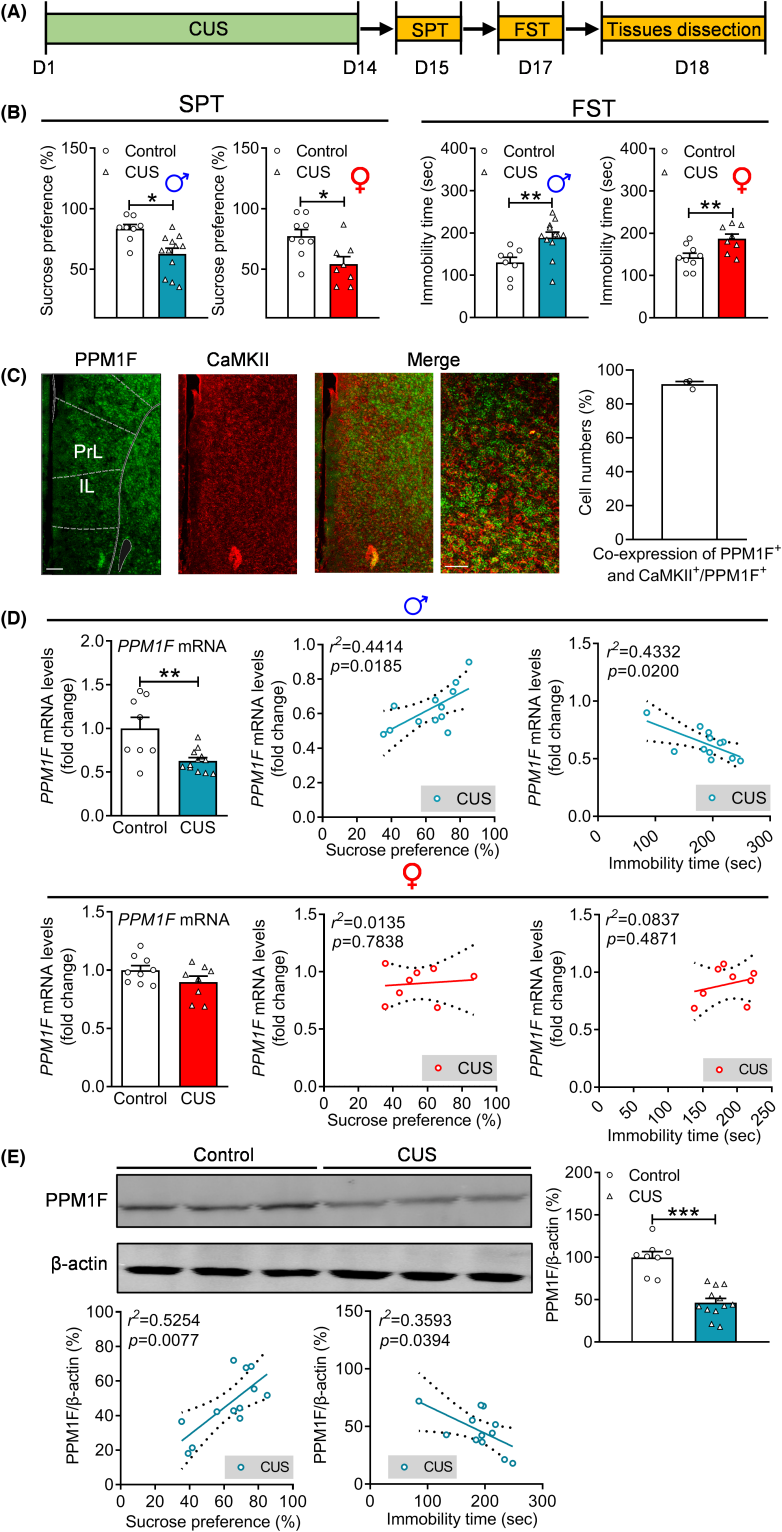
The downregulation of PPM1F expression in the mPFC by chronic unpredictable stress (CUS). (A) Schematic of the experimental designs. (B) Sucrose preference test, left: male mice; right: female mice. Forced swim test, left: male mice; right: female mice. (C) The representative image showing the co‐expression of PPM1F with CaMKII and quantitative analysis of percentage of PPM1F and CaMKII coexpression neurons with total PPM1F labeled neurons. Scale bar is 100 μm in low magnification and 25 μm in high magnification. *n* = 10 slices from 3 mice. (D) The mRNA expression levels of PPM1F in the mPFC of the control and CUS groups in both male and female mice, and the correlation analysis between the sucrose preference test, forced swim test, and PPM1F mRNA levels in CUS‐treated mice. Upper panel: male mice; lower panel: female mice. (E) PPM1F protein levels of control and CUS groups, and the correlation analysis between the sucrose preference test, forced swim test, and PPM1F protein levels in CUS treated mice. Male: control group, *n* = 8, CUS group, *n* = 12, female: control group, *n* = 9, CUS group, *n* = 8. *p < 0.05, **p < 0.01, ***p < 0.001 compared to the control group.

We aimed to explore whether PPM1F in the mPFC participates in the pathological process of depression. Therefore, PPM1F expression profiles were measured in the mPFC of depressed mice that showed obvious depressive behaviors after experiencing CUS according to our previous studies,^12^, ^28^ (Figure 1A), with reduced sucrose preference in the SPT (male: *t*_(18)_ = 3.1810, *p* = 0.0052; female: *t*_(15)_ = 2.7890, *p* = 0.0137) and increased immobility time in the FST (male: *t*_(18)_ = 3.1990, *p* = 0.0050; female: *t*_(15)_ = 2.9530, *p* = 0.0099; Figure 1B). First, regarding the distribution characteristics of PPM1F in the mPFC, we found that most PPM1F was coexpressed with excitatory neurons (91.6900 ± 1.5250%; Figure 1C), while PPM1F was rarely distributed in GABAergic (2.4670 ± 0.3844%) neurons or gliocyte (2.4000 ± 0.5686%; Figure 1C, Figure S1A,B). Moreover, our results indicated that PPM1F mRNA dramatically decreased in the mPFC of male, but not female depressed mice (male: *t*_(18)_ = 3.3300, *p* = 0.0037; female: *t*_(15)_ = 1.6050, *p* = 0.1294), and PPM1F mRNA levels were positively correlated with depression‐related behaviors, percentage of sucrose preference and immobility time in male mice only (Figure 1D). Furthermore, the reduced protein levels of PPM1F in the mPFC was only found in male, but not female mice (male: *t*_(18)_ = 6.4450, *p* < 0.0010; female: *t*_(15)_ = 0.4379, *p* = 0.6677; Figure 1E, Figure S1C), which was also positively correlated with depression‐related behaviors (Figure 1E).

### 3.2 Appearance of depression‐related behaviors induced by PPM1F knockdown

**FIGURE 2:**
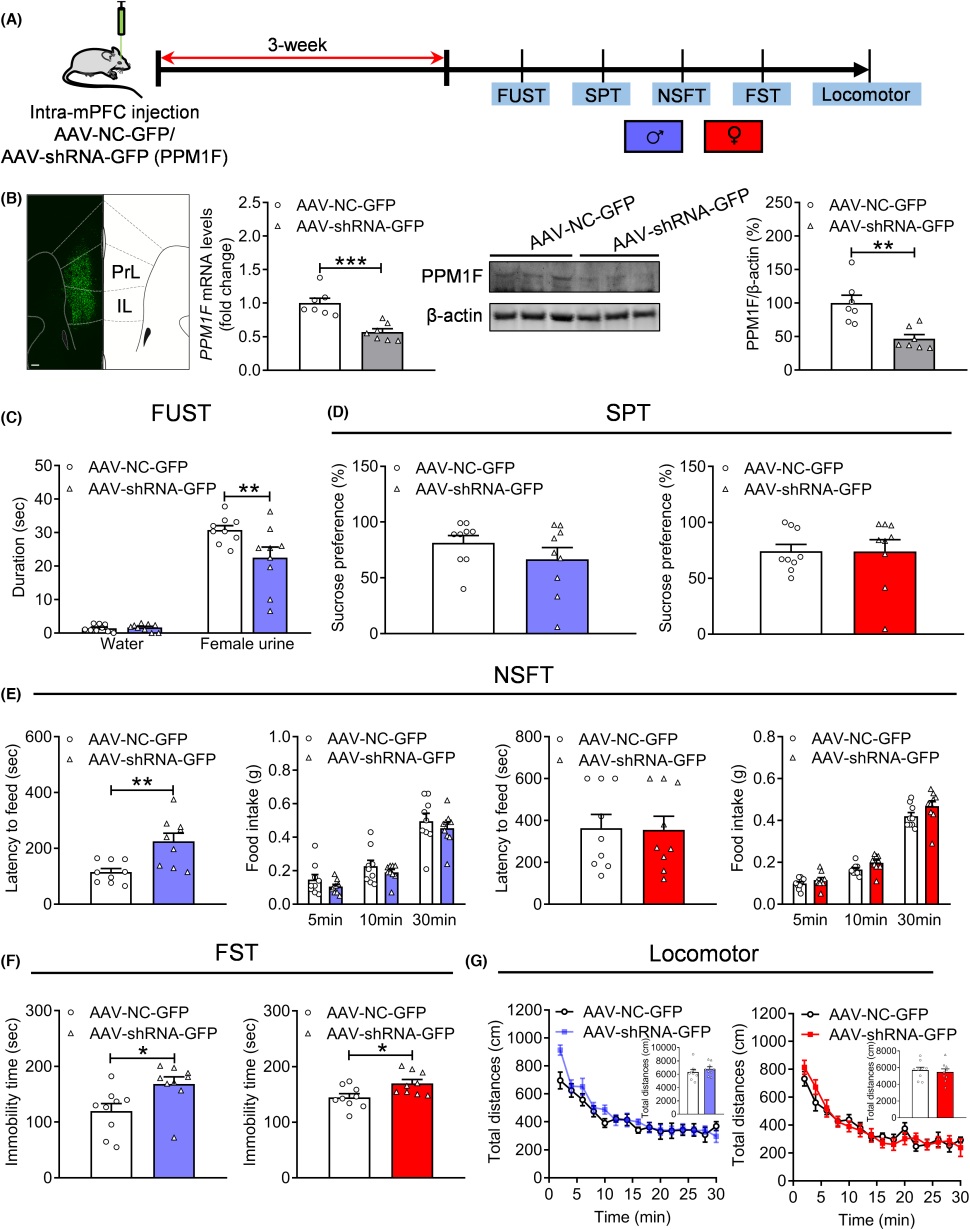
Induction of depression‐related behaviors by PPM1F knockdown in the mPFC. (A) Schematic diagram of the experimental timeline and depression‐related behaviors evaluation. (B) Left, A representative immunofluorescence image showing the expression of shRNA‐GFP in the mPFC. Scale bar is 100 μm. Middle and right, Real‐time PCR and western blot demonstrating the mRNA and protein expression level of PPM1F in the mPFC of AAV‐shRNA‐GFP injected mice. (C) Female urine sniffing test. (D) Sucrose preference test, left: male mice; right: female mice. (E) Novelty suppressed food test, left panel: male mice; right panel: female mice. (F) Forced swim test, left: male mice; right: female mice. (G) Locomotor activity, left panel: male mice; right panel: female mice. Male: AAV‐NC‐GFP group, *n* = 9, AAV‐shRNA‐GFP group, *n* = 9, female: AAV‐NC‐GFP group, *n* = 9, AAV‐shRNA‐GFP group, *n* = 9. *p < 0.05, **p < 0.01, ***p < 0.001, compared to the AAV‐NC‐GFP control group.

To examine the role of PPM1F in depression, we packaged an adeno‐associated virus mediating PPM1F knockdown using a short hairpin RNA (AAV‐shRNA‐GFP) strategy under the CaMKII promoter, which specifically targets excitatory neurons.^33^ Then, this virus was infused into the mPFC, and depression‐related behaviors were measured after 3 weeks (Figure 2A). Fluorescence analysis revealed that the expression area of AAV‐shRNA‐GFP was limited in the mPFC, while the specificity and efficiency of the PPM1F targeting shRNA, which has been previously demonstrated,^12^ was tested again to show that mRNA (*t*_(12)_ = 4.8750, *p* < 0.0010) and protein (*t*_(12)_ = 3.9740, *p* = 0.0018) levels were both significantly decreased compared to the control AAV‐NC‐GFP group (Figure 2B). Furthermore, we found that the sniffing time for water was nearly the same (*p* > 0.9999), but significantly decreased for female urine of the male AAV‐shRNA‐GFP mice (*p* = 0.0040; Figure 2C, treatment: *F*_(1,16)_ = 5.2420, *p* = 0.0360; sniffing object: *F*_(1,16)_ = 218.3000, *p* < 0.0010; treatment× sniffing object, *F*_(1,16)_ = 6.1440, *p* = 0.0247). Sucrose preference remained unchanged in both male and female AAV‐shRNA‐GFP mice, compared with the control AAV‐NC‐GFP group (Figure 2D, male: Mann–Whitney U test, *p* = 0.3278; female: Mann–Whitney U test, *p* = 0.6048). In the NSFT, there was an increased latency to food only in male AAV‐shRNA‐GFP mice (male: *t*_(16)_ = 3.4580, *p* = 0.0032; female: *t*_(16)_ = 0.0876, *p* = 0.9313), and none compared changes in the food intake of both male and female mice (Figure 2E, male: treatment: *F*_(1,16)_ = 1.0150, *p* = 0.3287; timepoint: *F*_(2,32)_ = 212.6000, *p* < 0.0010; treatment × timepoint, *F*_(2,32)_ = 0.0165, *p* = 0.9837; female: treatment: *F*_(1,16)_ = 3.5190, *p* = 0.0790; timepoint: *F*_(2,32)_414.5000, *p* < 0.0010; treatment × timepoint, *F*_(2,32)_ = 0.8582, *p* = 0.4334). Moreover, immobility time in the FST was greatly increased in both male and female AAV‐shRNA‐GFP mice compared with the control AAV‐NC‐GFP group (Figure 2F, male: Mann–Whitney U test, *p* = 0.0106; female: *t*_(16)_ = 2.6420, *p* = 0.0178). Finally, we evaluated the locomotor activity of these mice and found no significant changes (Figure 2G, male: treatment: *F*_(1,16)_ = 0.9105, *p* = 0.3542; timepoint: *F*_(14,224)_ = 34.6300, *p* < 0.0010; treatment × timepoint, *F*_(14,224)_ = 1.8970, *p* = 0.0278; total distance: *t*_(16)_ = 0.9542, *p* = 0.3542; female: treatment: *F*_(1,16)_ = 0.0003, p = 0.9873; timepoint: F_(14, 224)_ = 42.2500, *p* < 0.0010; treatment × timepoint, *F*_(14,224)_ = 1.3210, *p* = 0.1960; total distance: *t*_(16)_ = 0.4319, *p* = 0.6716), indicating that the behavioral alterations in PPM1F knockdown mice were not caused by a change in spontaneous locomotor activity, but rather by depression‐related behavioral changes.

Moreover, we measured anxiety‐related behaviors (Figure S2A) and found that female, but not male, AAV‐shRNA‐GFP mice showed lower percentage of open arm time (male: *t*_(16)_ = 0.1987, *p* = 0.8450; female: *t*_(16)_ = 3.2350, *p* = 0.0052), and open arm entries (male: *t*_(16)_ = 0.6249, *p* = 0.5408; female: *t*_(16)_ = 2.2580, *p* = 0.0383), and both male and female mice showed approximately similar total arm entries (*t*_(16)_ = 0.5230, *p* = 0.6081; female: *t*_(16)_ = 1.1830, *p* = 0.2542) compared to control AAV‐NC‐GFP‐injected mice in the EPM test (Figure S2B). We also found that male and female AAV‐shRNA‐GFP‐injected mice showed no differences in the center time (male: *t*_(16)_ = 0.7749, *p* = 0.4497; female: *t*_(16)_ = 0.5068, *p* = 0.6192) or total distance (male: *t*_(16)_ = 1.1890, *p* = 0.2518; female: *t*_(16)_ = 0.9853, *p* = 0.3391) compared to AAV‐NC‐GFP‐injected mice in the OFT (Figure S2C).

### 3.3 Overexpression of PPM1F produces an antidepressant effect in depressed mice

**FIGURE 3:**
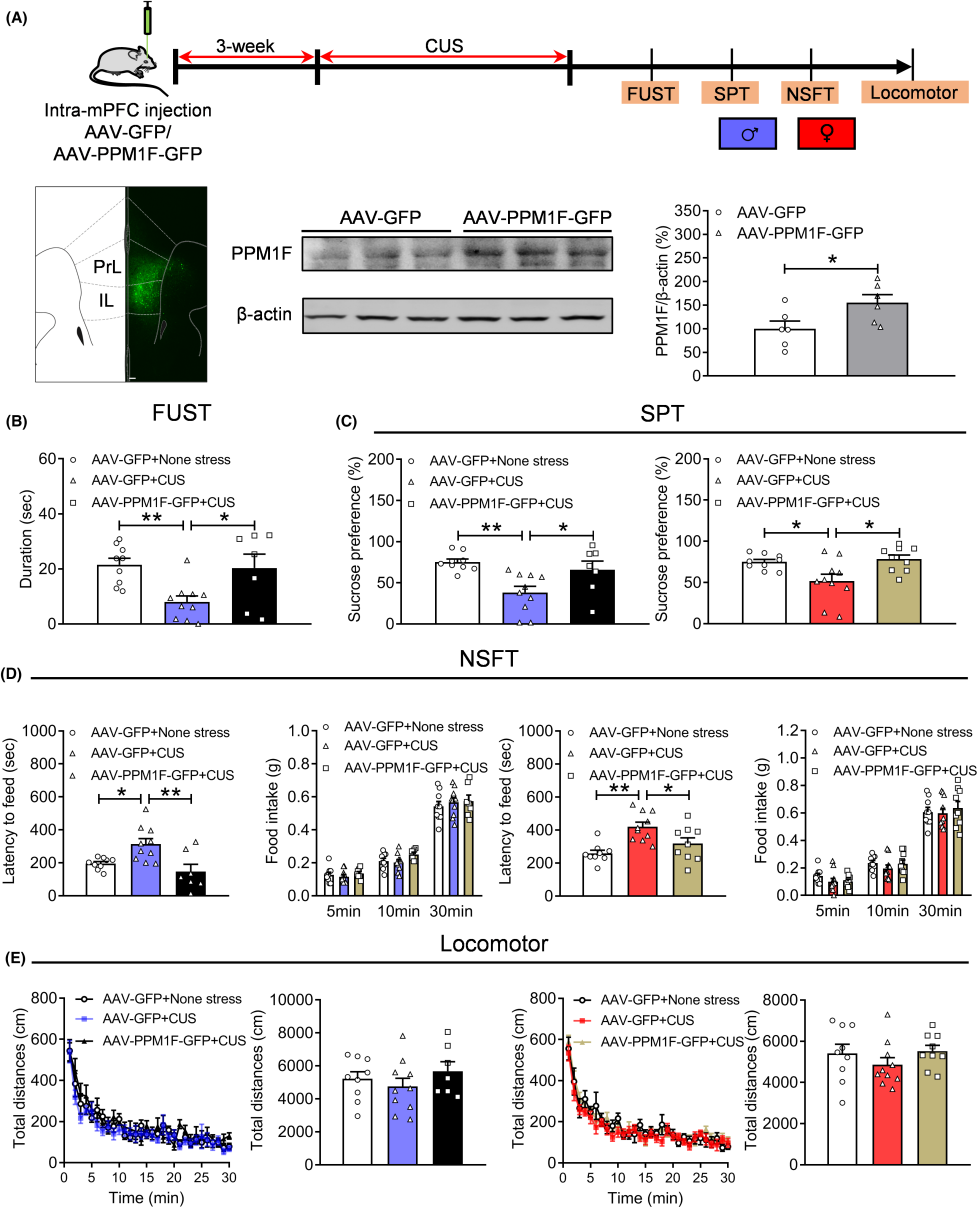
Overexpression of PPM1F in the mPFC produces the anti‐depressive activity under stress conditions. (A) Upper panel, schematic diagram of the experimental timeline. Lower‐left, a representative immunofluorescence image showing the expression of AAV‐PPM1F‐GFP in the mPFC. Scale bar is 100 μm. Lower‐right panel, the protein expression levels of PPM1F in the mPFC of AAV‐PPM1F‐GFP‐injected mice. AAV‐GFP: *n* = 6, AAV‐PPM1F‐GFP group, *n* = 6. (B) Female urine sniffing test. (C) Sucrose preference test, left: male mice; right: female mice. (D) Novelty suppressed food test, left panel: male mice; right panel: female mice. (E) Locomotor activity, left: male mice; right: female mice. Male: AAV‐GFP + None stress group: *n* = 9, AAV‐GFP + CUS group, *n* = 10, AAV‐PPM1F‐GFP + CUS group, *n* = 7, female: AAV‐GFP + None stress group: *n* = 9, AAV‐GFP + CUS group, *n* = 10, AAV‐PPM1F‐GFP + CUS group, *n* = 9. *p < 0.05, **p < 0.01, compared to the control group.

Next, we packaged adeno‐associated virus that mediates PPM1F overexpression under the CaMKII promoter. Depression‐related behaviors were measured after injecting this virus into the mPFC (Figure S3A). We observed that GFP fluorescence was concentrated in the mPFC, and protein (*t*_(10)_ = 2.3570, *p* = 0.0402) levels were increased in the AAV‐PPM1F‐GFP group compared with the control AAV‐GFP group (Figure 3A). We also found that the sniffing times for both water (*p* > 0.9999) and female urine (*p* = 0.1968) were similar to those of male AAV‐GFP mice (Figure S3B, treatment: *F*_(1,17)_ = 1.2830, *p* = 0.2730; sniffing object: *F*_(1,17)_ = 131.5000, *p* < 0.0010; treatment × sniffing object, *F*_(1,17)_ = 16080, *p* = 0.2219). We observed no clear differences for sucrose preference (Figure S3C, male: *t*_(17)_ = 0.0881, *p* = 0.9308; female: *t*_(18)_ = 0.9349, *p* = 0.3622) in the SPT, or the latency to food (male: *t*_(17)_ = 0.8428, *p* = 0.4111; female: *t*_(18)_ = 0.5251, *p* = 0.6059) and food intake (Figure S3D, male: genotype: *F*_(1,17)_ = 0.6701, *p* = 0.4244; timepoint: *F*_(2,34)_ = 251.1000, *p* < 0.0010; genotype × timepoint, *F*_(2,34)_ = 1.4460, *p* = 0.2496; female: genotype: *F*_(1,18)_ = 0.0164, *p* = 0.8994; timepoint: *F*_(2,36)_ = 437.7000, *p* < 0.0010; genotype × timepoint, *F*_(2,36)_ = 0.3183, *p* = 0.7249) in both male and female AAV‐PPM1F‐GFP‐treated mice in the NSFT. In contrast, immobility time in the FST was decreased only in male AAV‐PPM1F‐GFP mice compared with the control AAV‐GFP mice (Figure S3E, male: *t*_(17)_ = 2.3220, *p* = 0.0329; female: *t*_(18)_ = 0.4423, *p* = 0.6635). Finally, the locomotor activity test results showed no significant changes in these groups (Figure S3F, male: treatment: *F*_(1,17)_ = 0.7736, *p* = 0.3914; timepoint: *F*_(29,493)_ = 26.5100, *p* < 0.0010; treatment × timepoint, *F*_(29,493)_ = 0.5350, *p* = 0.9788; total distance: Mann–Whitney U test, *p* = 0.3562; female: treatment: *F*_(1,18)_ = 1.0580, *p* = 0.3173; timepoint: *F*_(29,522)_ = 23.1600, *p* < 0.0010; treatment × timepoint, *F*_(29,522)_ = 1.0130, *p* = 0.4490; total distance: *t*_(18)_ = 1.0280, *p* = 0.3173).

We also evaluated anxiety‐related behaviors (Figure S4A), and a higher percentage of open arm time (male: *t*_(17)_ = 2.5960, *p* = 0.0188; female: *t*_(18)_ = 1.5190, *p* = 0.1462) and open arm entries (male: *t*_(17)_ = 2.2880, *p* = 0.0352; female: Mann–Whitney U test, *p* = 0.4683) were only observed in male AAV‐PPM1F‐GFP mice compared with AAV‐GFP control mice. The total arm entries of both male and female (male: *t*_(17)_ = 1.2690, *p* = 0.2216; female: *t*_(18)_ = 0.1758, *p* = 0.8624) AAV‐PPM1F‐GFP mice remained unchanged compared with AAV‐GFP‐injected mice in the EPM test (Figure S4B). We also found that the time in the center (male: *t*_(17)_ = 0.8248, *p* = 0.4209; female: Mann–Whitney U test, *p* = 0.5787) and total distance (male: *t*_(17)_ = 1.2320, *p* = 0.2349; female: *t*_(18)_ = 0.4675, *p* = 0.6457) were not affected for either male or female AAV‐PPM1F‐GFP‐injected mice compared with AAV‐GFP‐injected mice in the OFT (Figure S4C).

Furthermore, we measured the effects of PPM1F overexpression in the mPFC on depression‐related behaviors under stress conditions by subjecting the mice to CUS (Figure 3A). The results showed that CUS significantly reduced sniffing time for female urine (*p* = 0.0093), which was consistent with our previous reports,^28^, ^33^ and that PPM1F overexpression can rescue this decline (*p* = 0.0283; Figure 3B, *F*_(2,23)_ = 6.4050, *p* = 0.0061). Other depressive phenotypes induced by CUS, such as reduced sucrose preference (male: *p* = 0.0031; female: *p* = 0.0302) and increased latency (male: *p* = 0.0263; female: *p* = 0.0011) to food, were also reversed by PPM1F overexpression (Figure 3C, sucrose preference, male: *p* = 0.0416, female: *p* = 0.0302; Figure 3D, latency: male: *p* = 0.0033, female: *p* = 0.0398) in both male (SPT: *F*_(2,23)_ = 7.5000, *p* = 0.0031; NSFT: *F*_(2,23)_ = 7.6730, *p* = 0.0028) and female (SPT: *F*_(2,25)_ = 5.8750, *p* = 0.0081; NSFT: *F*_(2,25)_ = 8.6490, *p* = 0.0014) mice, while food intake remained unchanged (Figure 3D, male: genotype: *F*_(2,23)_ = 0.6318, *p* = 0.5406; timepoint: *F*_(2,46)_ = 714.9000, *p* < 0.0010; genotype × timepoint, *F*_(4,46)_ = 0.8874, *p* = 0.4790; female: *F*_(2,25)_ = 0.4526, *p* = 0.6411; timepoint: *F*_(2,50)_ = 724.8000, *p* < 0.0010; genotype × timepoint, *F*_(4,50)_ = 1.1280, *p* = 0.3540). Finally, we observed no significant differences in these groups for locomotor activity (Figure 3E, male: treatment: *F*_(2,23)_ = 0.8334, *p* = 0.4473; timepoint: *F*_(29,667)_ = 35.4600, *p* < 0.0010; treatment × timepoint, *F*_(58,667)_ = 0.9060, *p* = 0.6729; total distance: *F*_(2,23)_ = 0.8334, *p* = 0.4473; female: treatment: *F*_(2,25)_ = 0.9833, *p* = 0.3881; timepoint: *F*_(29,725)_ = 48.5100, *p* < 0.0010; treatment × timepoint, *F*_(58,725)_ = 0.8106, *p* = 0.8414; total distance: *F*_(2,25)_ = 0.9833, *p* = 0.3881).

### 3.4 PPM1F knockdown decreased the excitability of pyramidal neurons in the mPFC

**FIGURE 4:**
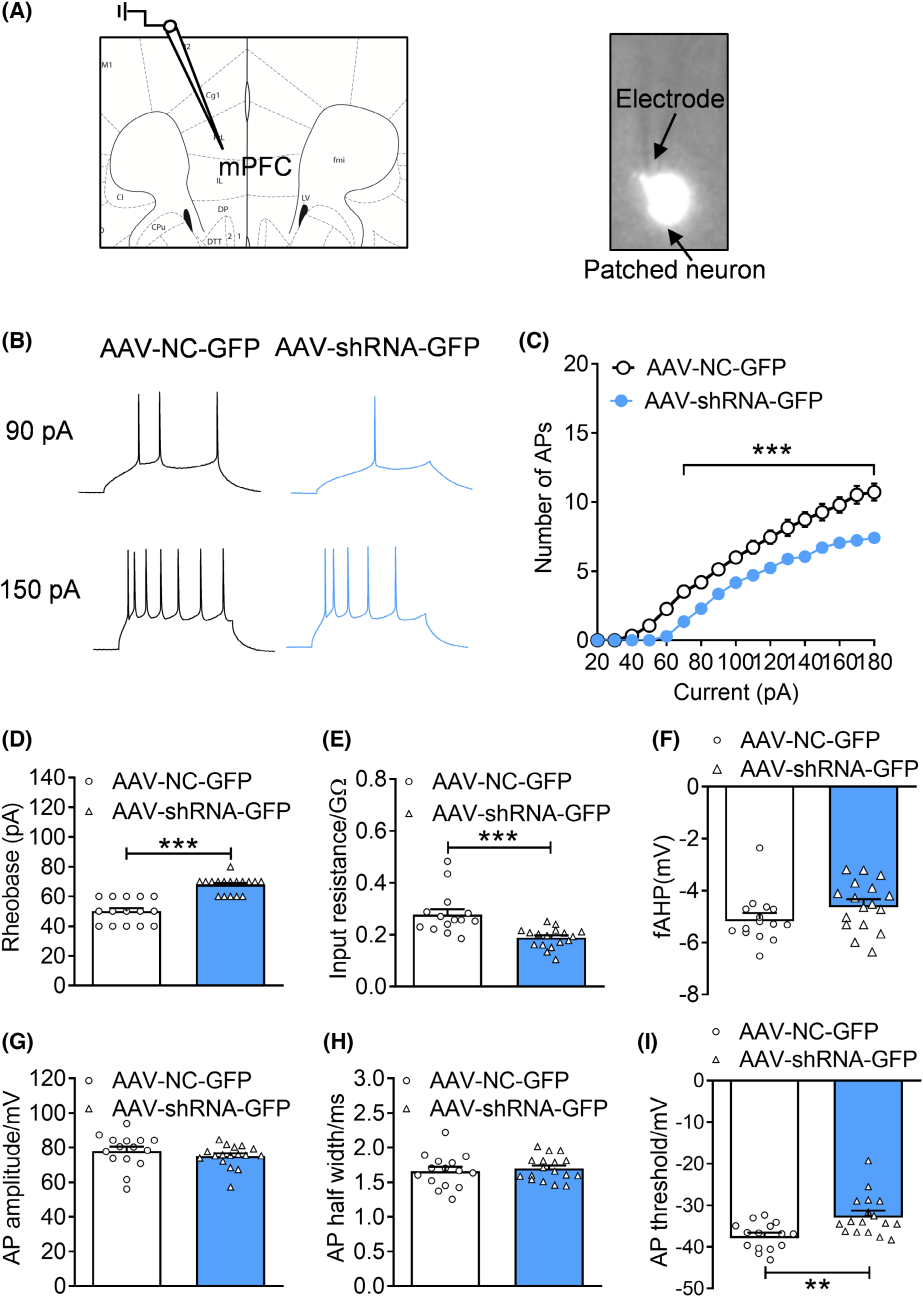
PPM1F knockdown in the mPFC decreases the neuronal excitability of the pyramidal cells. (A) Schematic illustration of recordings from pyramidal cells in the mPFC brain slices. (B) Depolarizing current injections evoke trains of action potentials (AP) in mPFC neurons. (C) Number of AP elicited by depolarizing current injection. (D) Rheobase current. (E) Input resistance. (F) fAHP amplitude. (G) Amplitude of AP. (H) AP half‐width. (I) AP threshold. AAV‐NC‐GFP group, *n* = 15 neurons from 3 mice, AAV‐shRNA‐GFP group, *n* = 17 neurons from 4 mice. **p < 0.01, ***p < 0.001, compared to the AAV‐NC‐GFP control group.

Electrophysiological dysfunction is a pivotal characteristic of the pathophysiological process of depression. Therefore, we recorded the excitability of neurons in the mPFC after PPM1F knockdown (Figure 4A). The results showed fewer action potentials in response to the same amount of current injections (Figure 4B,C, current: *F*_(17,510)_ = 465.8000, *p* < 0.0010; treatment: *F*_(1,30)_ = 32.2000, *p* < 0.0010; current × treatment: *F*_(17,510)_ = 11.6200, *p* < 0.0010), higher rheobase current (Figure 4D, Mann–Whitney U test, *p* < 0.0010), and lower input resistance (Figure 4E, Mann–Whitney U test, *p* < 0.0010) were found in the neurons of AAV‐shRNA‐GFP mice than in those of AAV‐NC‐GFP mice. Furthermore, the fAHP amplitude (Figure 4F, Mann–Whitney U test, *p* = 0.0757), amplitude (Figure 4G, *t*_(30)_ = 0.9921, *p* = 0.3291) and half‐width of action potentials (Figure 4H, *t*_(30)_ = 0.5161, *p* = 0.6096) were similar between the two groups, whereas the action potential threshold decreased in the AAV‐shRNA‐GFP mice compared with the AAV‐NC‐GFP mice (Figure 4I, Mann–Whitney U test, *p* = 0.0017).

To explore the molecular mechanisms of PPM1F modulation of the reduced excitability, we measured the mRNA expression of α‐amino‐3‐hydroxy‐5‐methyl‐4‐isoxazolepropionic acid (AMPA) receptor, N‐methyl‐D‐aspartic acid (NMDA) receptor and some K+ channel genes. The results showed that PPM1F knockdown have no effects on the mRNA levels of AMPA (GluR1: Mann–Whitney test, *p* = 0.4848, GluR2: *t*_(10)_ = 0.5884, *p* = 0.5693, GluR3: *t*_(10)_ = 1.0190, *p* = 0.3322) and NMDA receptors (NR1: *t*_(10)_ = 0.7478, *p* = 0.4718, NR2A: *t*_(10)_ = 0.0574, *p* = 0.9554, NR2B: *t*_(10)_ = 0.9188, *p* = 0.3798; Figure S5A,B), but decreased the the mRNA levels of kcnn1 (*t*_(10)_ = = 2.4050, *p* = 0.0370) and kcnj2 (*t*_(10)_ = 2.5560, *p* = 0.0286), not kcnf1 (*t*_(10)_ = 0.6792, *p* = 0.5124) (Figure S5C).

### 3.5 Counteracting the reduced excitability of pyramidal neurons restores the depression‐related behaviors induced by PPM1F knockdown

**FIGURE 5:**
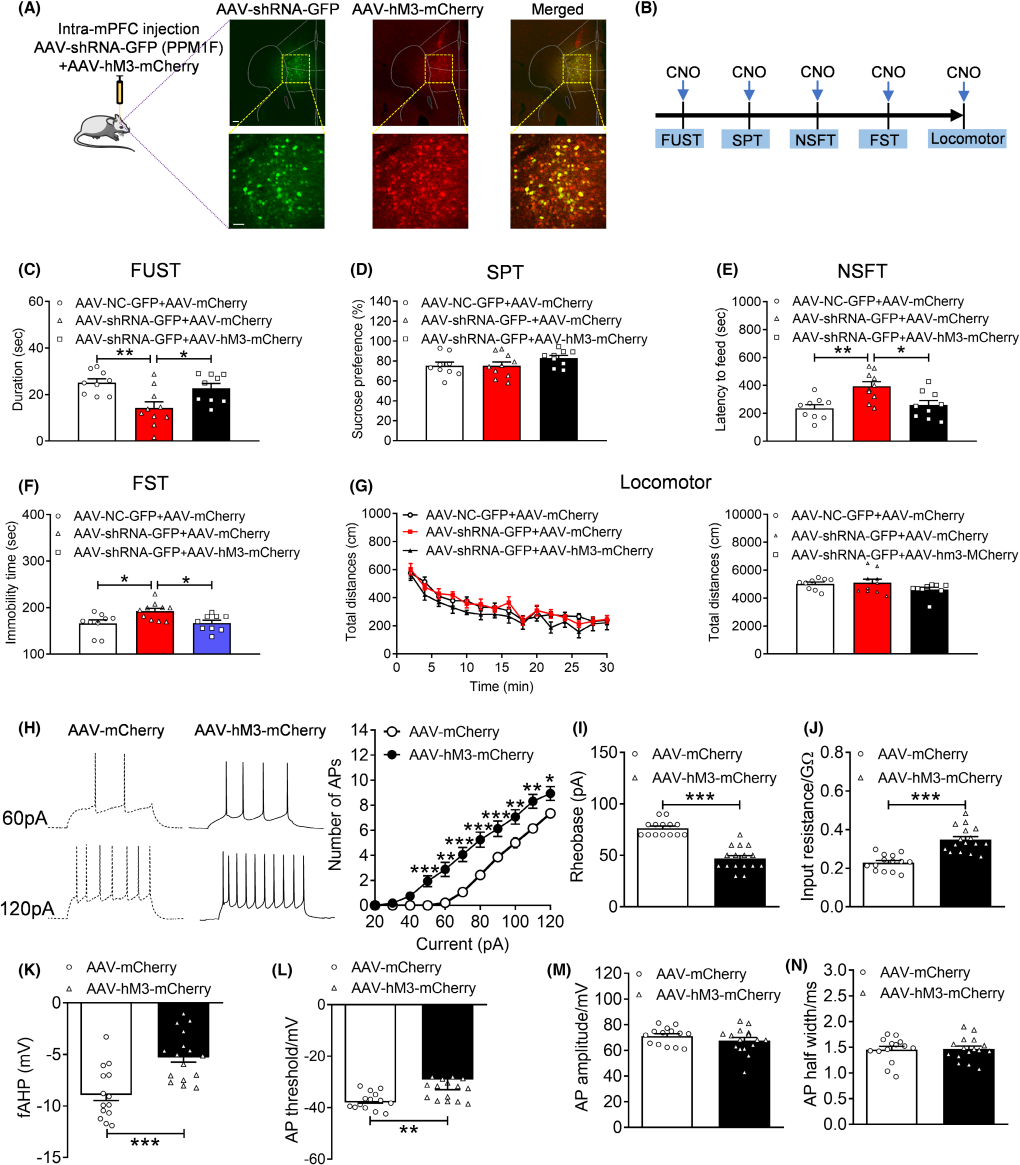
The effect of promoting the excitability of the excitatory neurons on the depressive phenotypes induced by PPM1F knockdown. (A). Representative image showing the coexpression profile of AAV‐shRNA‐GFP and AAV‐hM3‐mCherry in the mPFC. Scale bar is 100 μm in low magnification and 25 μm in high magnification. (B) Schematic of the experiment. (C) Female urine sniffing test with CNO injection. (D) Sucrose preference test with CNO injection. (E) Novelty suppressed food test with CNO injection. (F) Forced swim test with CNO injection. (G) Locomotor activity with CNO injection. AAV‐NC‐GFP + AAV‐mCherry group, *n* = 9, AAV‐shRNA‐GFP + AAV‐mCherry group, *n* = 10, AAV‐shRNA‐GFP + AAV‐hM3‐mCherry group, *n* = 9. (H) Number of AP elicited by depolarizing current injection. (I) Rheobase current. (J) Input resistance. (K) fAHP amplitude. (L) AP threshold. (M) Amplitude of AP. (N) AP half‐width. AAV‐mCherry group, *n* = 14 neurons from 3 mice, AAV‐hM3‐mCherry group, *n* = 16 neurons from 3 mice. *p < 0.05, **p < 0.01, ***p < 0.001 compared to the control group.

Next, we used a chemogenetic approach to determine whether the activation of blocked neuronal excitability of the neurons in the mPFC induced by PPM1F knockdown was sufficient to reverse depression‐related behaviors. We infused the mixtures of AAV‐shRNA‐GFP, AAV‐NC‐GFP, AAV‐hM3D‐mCherry or AAV‐mCherry viruses into the mPFC bilaterally, and then injected clozapine‐N‐oxide (CNO) to activate neuronal excitability of the neurons in the mPFC. After finding that the coinjected viruses were robustly coexpressed in the mPFC, we evaluated the depression‐related behaviors (Figure 5A,B). The results showed that AAV‐hM3‐mCherry mediating neural activation prevented decreases in sniffing time for female urine (*p* = 0.0270) induced by the AAV‐shRNA‐GFP treatment (Figure 5C, *p* = 0.0043; *F*_(2,25)_ = 7.0670, *p* = 0.0037). In contrast, we observed no differences in sucrose preference after PPM1F knockdown or neuronal excitability activation (Figure 5D, *F*_(2,25)_ = 1.6470, *p* = 0.2129). Moreover, the increased latency to food in the NSFT (*p* = 0.0033) and increased immobility in the FST (*p* = 0.0423) generated by PPM1F knockdown were also inhibited by AAV‐hM3‐mCherry mediating neural activation (Figure 5E,F, *p* = 0.0123 and *p* = 0.0389, respectively; NSFT: *F*_(2,25)_ = 7.9160, *p* = 0.0022; FST: Kruskal–Wallis test, *p* = 0.0159). Furthermore, we found no significant genotype treatment (*F*_(2,25)_ = 2.8490, *p* = 0.0768) or timepoint effects on total distance (*F*_(14,350)_ = 34.5900, *p* < 0.0010), without an interaction effect between treatment and timepoint (*F*_(28,350)_ = 0.5993, *p* = 0.9487). We also observed no significant between group difference in total distance (Figure 5G, Kruskal–Wallis test, *p* = 0.3265).

The efficiency of the chemogenetic approach was also demonstrated, and the results indicated that an elevated number of action potentials in response to the same amount of current injections (Figure 5H, current: *F*_(11,308)_ = 275.4000, *p* < 0.0010; treatment: *F*_(1,28)_ = 14.6500, *p* < 0.0010; current × treatment: *F*_(11,308)_ = 9.4740, *p* < 0.0010), decreased rheobase current (Figure 5I, Mann–Whitney U test, *p* < 0.0010), increased input resistance (Figure 5J, *t*_(28)_ = 5.8350, *p* < 0.0010), decreased fAHP amplitude (Figure 5K, *t*_(28)_ = 4.0330, *p* < 0.0010) and action potentials threshold (Figure 5L, Mann–Whitney U test, *p* = 0.0024) were found in the neurons of AAV‐hM3‐mCherry mice compared with the control AAV‐mCherry mice. Nevertheless, amplitude (Figure 5M, *t*_(28)_ = 1.1200, *p* = 0.2721) and half‐width of action potentials (Figure 5N, *t*_(28)_ = 0.1463, *p* = 0.8847) were similar between both groups.

### 3.6 PPM1F regulated p300 expression by modifying AMPK activity

**FIGURE 6:**
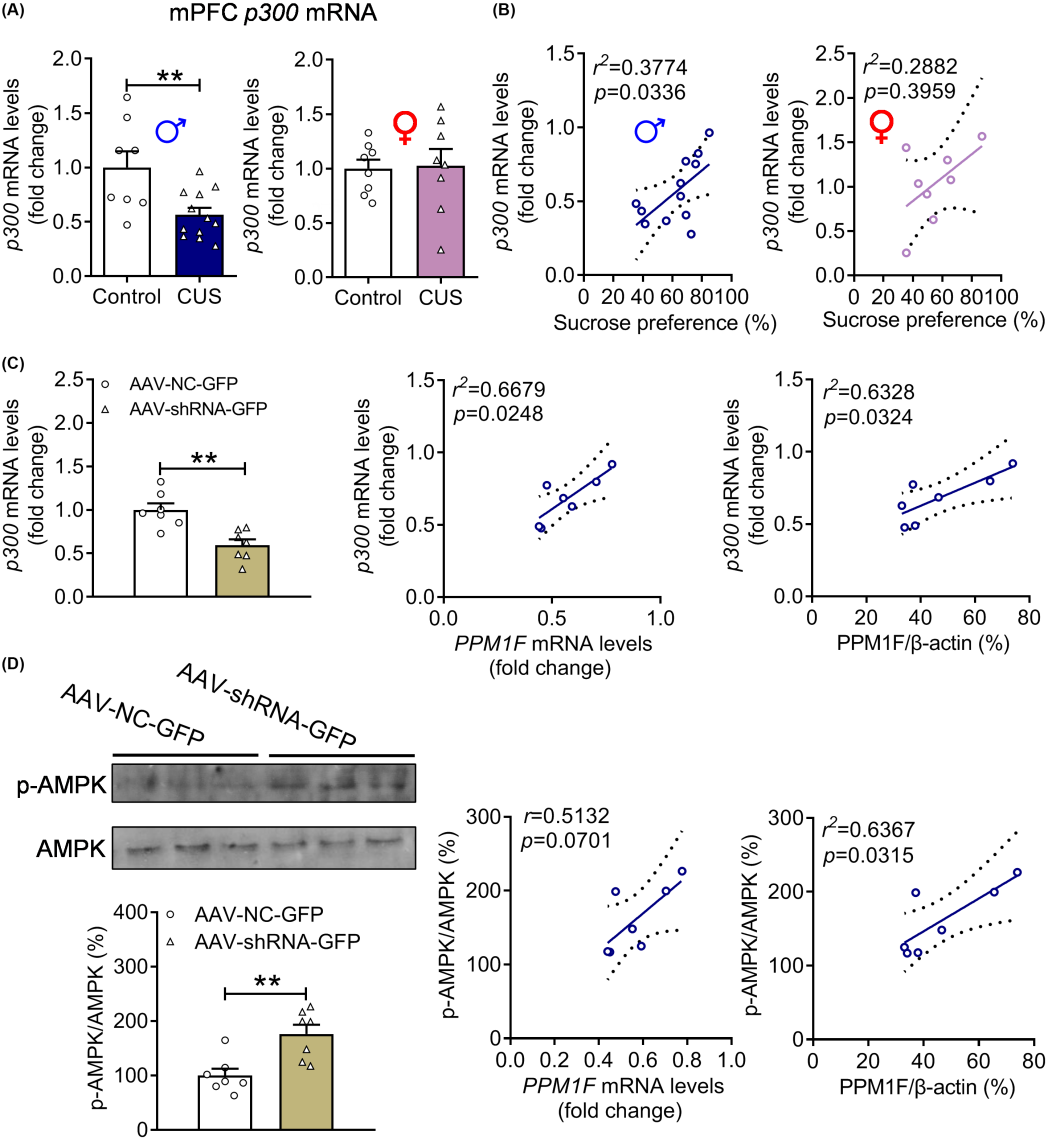
The regulation of expression levels of p300 and activation of AMPK in the mPFC of PPM1F knockdown mice. (A) The mRNA levels of p300 in mPFC of CUS mice. Male: control group, *n* = 8, CUS group, *n* = 12; female: control group, *n* = 8, CUS group, *n* = 8. (B) The correlation analysis between p300 mRNA levels and sucrose preference. (C) The mRNA levels of p300 in mPFC of PPM1F knockdown mice, and the correlation analysis of PPM1F mRNA or protein levels with p300 mRNA levels. (D) The phosphorylation of AMPK levels and the correlation analysis between PPM1F levels and phosphorylation of AMPK levels. AAV‐NC‐GFP group, *n* = 7, AAV‐shRNA‐GFP group, *n* = 7. **p < 0.01, compared to the control group.

To verify the role of p300 in depression, we first measured the expression levels of p300 in the mPFC of depressed male and female mice, and found that CUS decreased the mRNA levels of p300 only in male mice (Figure 6A, male: *t*_(18)_ = 3.0580, *p* = 0.0068; female: *t*_(14)_ = 0.1554, *p* = 0.8787). Furthermore, only the p300 mRNA levels of male mice were positively correlated with depression‐related behaviors, such as the sucrose preference (Figure 6B). We then tested whether p300 expression was regulated by PPM1F, and observed that PPM1F knockdown significantly decreased the mRNA levels of p300 (Figure 6C *t*_(12)_ = 4.0400, *p* = 0.0016), and p300 mRNA levels were positively correlated with the mRNA or protein levels of PPM1F (Figure 6C). Previous research has reported that AMPK is a downstream signaling pathway of PPM1F,^12^ and the activity of p300 is inhibited by AMPK mediating phosphorylation.^34^ Therefore, we measured the effect of PPM1F on the activity of AMPK and found that the phosphorylation levels of AMPK were obviously increased by PPM1F knockdown (*t*_(12)_ = 3.6230, *p* = 0.0035) and positively correlated with the mRNA and protein levels of PPM1F (Figure 6D).

Next, as many reports have suggested a significant association between neuroinflammation and depression,^35^, ^36^ and our previous research has implied that PPM1F may regulate the neuroinflammation reactivity,^37^ therefore, we investigated the neuroinflammation‐related molecules after PPM1F knockdown in the mPFC, we found that the number of iba1^+^ cells, a marker for microglia was increased in the PPM1F knockdown group (Figure S6A, *t*_(10)_ = 2.7850, *p* = 0.0193). Meanwhile, the levels of iNOS and Arg‐1, markers of microglia/macrophage polarization were measured, and the results indicated that the mRNA level of iNOS remains unchanged (*t*_(12)_ = 1.0870, *p* = 0.2986), while the mRNA level of Arg‐1 was decreased in the PPM1F knockdown group (*t*_(12)_ = 3.4010, *p* = 0.0053; Figure S6B). Moreover, the expression of proinflammatory markers TNF‐α (*t*_(12)_ = 2.5570, *p* = 0.0251) and IL‐6 (*t*_(12)_ = 2.5860, *p* = 0.0239), but not IL‐1β (*t*_(12)_ = 0.2528, *p* = 0.8047) was significantly upregulated, while the expression of the anti‐inflammatory markers IL‐4 (*t*_(12)_ = 0.3444, *p* = 0.7365) was not regulated in the PPM1F knockdown group compared to the control group (Figure S6B).

### 3.7 Knockout of AMPK produced an antidepressant activity under stress conditions

Based on the finding that PPM1F knockdown increased the phosphorylation levels of AMPK, which can produce depressive‐related behaviors, we assumed that knockout of AMPK may bear the antidepressant activity. Therefore, we first injected the AAV‐CaMKII‐GFP or AAV‐CaMKII‐Cre virus into the mPFC of AMPKα2 flox mice to knock out the α2 subunit of AMPK, which was the targeted phosphorylation site of AMPK (Figure S7A). The fluorescence results showed that the infused virus was specifically expressed in the mPFC, and the mRNA levels of exon2 of AMPK dramatically decreased (Figure S7B, (*t*_(10)_ = 2.6300, *p* = 0.0252). Next, we measured depression‐related behaviors without stress and found that AAV‐CaMKII‐Cre‐treated mice showed nearly the same sniffing time for water or female urine as AAV‐CaMKII‐GFP‐treated mice (Figure S7C, treatment: *F*_(1,16)_ = 0.1887, *p* = 0.6698; sniffing object: *F*_(1,16)_ = 86.7700, *p* < 0.0010; treatment × sniffing object, *F*_(1,16)_ = 0.0328, *p* = 0.8586), with no changes observed in sucrose preference for these two groups (Figure S7D, *t*_(16)_ = 1.0340, *p* = 0.3163). Furthermore, these mice were subjected to CUS, and their depression‐related behavior was tested under stress conditions. We found that AAV‐CaMKII‐Cre‐treated mice showed increased sniffing time for female urine (*p* = 0.0140), but not for water (*p* > 0.9999; Figure S7E, treatment: *F*_(1,16)_ = 5.4330, *p* = 0.0332; sniffing object: *F*_(1,16)_ = 40.5900, *p* < 0.0010; treatment × sniffing object, *F*_(1,16)_ = 5.5050, *p* = 0.0322), and increased sucrose preference (Figure S7F, Mann–Whitney U test, *p* = 0.0343) in the SPT compared with AAV‐CaMKII‐GFP‐treated mice. Nevertheless, latency to food (Figure S7G, Mann–Whitney U test, *p* = 0.9151) and food intake (treatment: *F*_(1,16)_ = 0.6767, *p* = 0.4228; timepoint: *F*_(2,32)_ = 188.5000, *p* < 0.0010; treatment × timepoint, *F*_(2,32)_ = 0.3852, *p* = 0.6835) did not differ between the two groups. Immobility time in the FST was reduced for the AAV‐CaMKII‐Cre‐treated mice (Figure S7H, *t*_(16)_ = 5.1720, *p* < 0.0010), whereas both groups showed nearly similar locomotor activity (Figure S7I, treatment: *F*_(1,16)_ = 0.0007, *p* = 0.9939; timepoint: *F*_(14,224)_ = 14.8100, *p* < 0.0010; treatment × timepoint, *F*_(14,224)_ = 1.5030, *p* = 0.1111; total distance: (*t*_(16)_ = 0.0078, *p* = 0.9939).

We also assessed anxiety‐related behaviors (Figure S8A), and observed that the percentage of open arm time (*t*_(16)_ = 0.7983, *p* = 0.4364), open arm entries (*t*_(16)_ = 1.4220, *p* = 0.1743), and total arm entries (*t*_(16)_ = 0.7061, *p* = 0.4903) were comparable between AAV‐CaMKII‐Cre and AAV‐CaMKII‐GFP control mice in the EPM test (Figure S8B). Moreover, we found that the time in the center (*t*_(16)_ = 1.0710, *p* = 0.2999) and total distance (*t*_(16)_ = 1.2770, *p* = 0.2199) did not differ in AAV‐CaMKII‐Cre‐treated mice compared with AAV‐CaMKII‐GFP mice in the OFT (Figure S8C).

### 3.8 AMPK knockout blocked depression‐related behaviors induced by PPM1F knockdown

**FIGURE 7:**
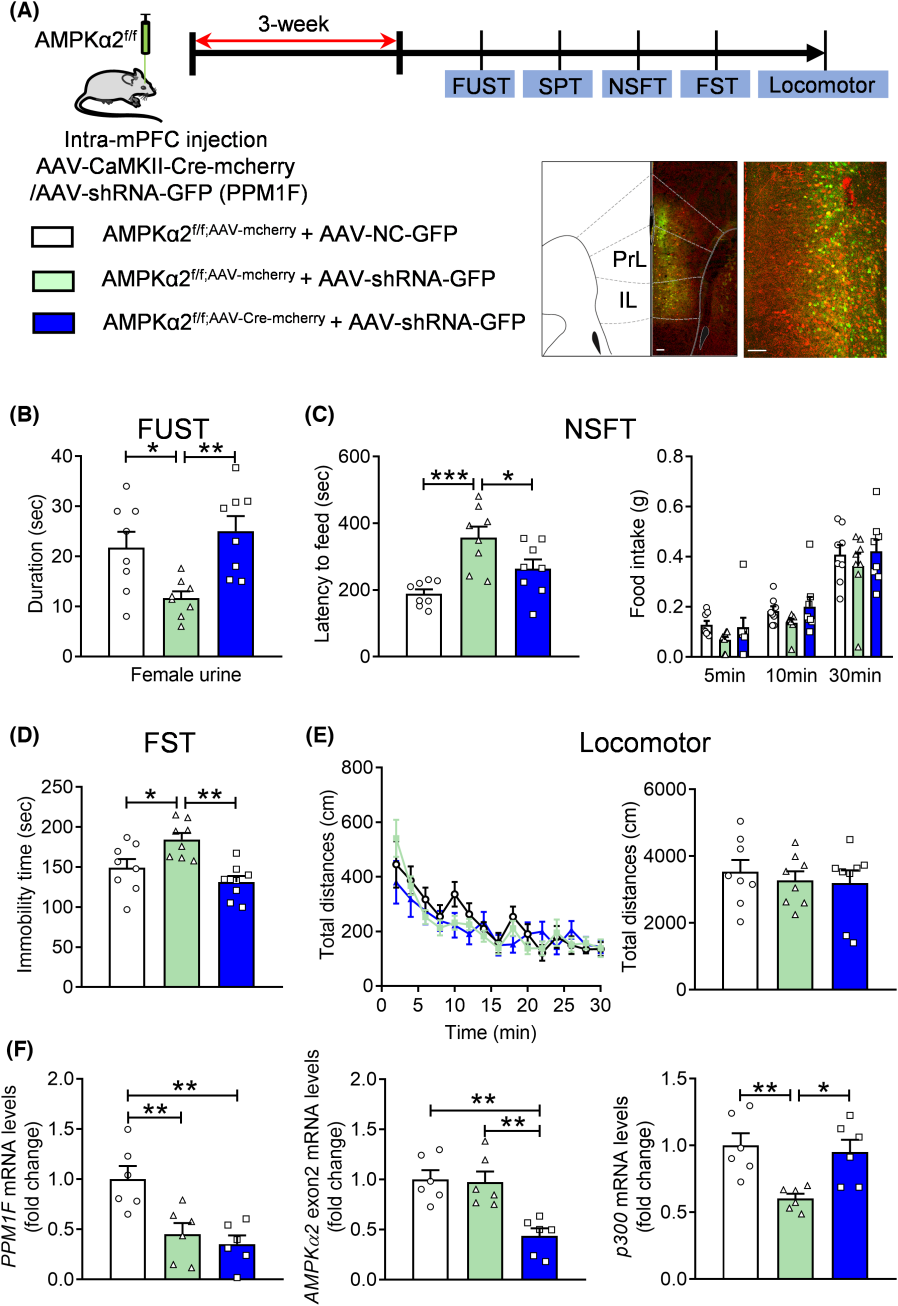
AMPK knockout suppresses the depression‐related behaviors induced by PPM1F knockdown. (A) Upper panel, schematic diagram of the experimental timeline. Lower panel, a representative immunofluorescence image showing the coexpression of AAV‐CaMKII‐Cre‐mcherry and AAV‐shRNA‐GFP in the mPFC of AMPKα2^f/f^ mice. Scale bar is 100 μm in low magnification and 25 μm in high magnification. (B) Female urine sniffing test. (C) Novelty suppressed food test. (D) Forced swim test. (E) Locomotor activity. *n* = 8 per group. (F) The mRNA levels of PPM1F, AMPKα2 exon2 and p300. *n* = 6 per group. *p < 0.05, **p < 0.01, ***p < 0.001, compared to the control group.

To identify whether AMPK is a pivotal and integral mediator in the relationship between PPM1F and depression, we further investigated whether abolishing AMPK activity by knockout of AMPK can contradict depression‐related behaviors in PPM1F knockdown mice. AAV‐CaMKII‐Cre‐mCherry and AAV‐shRNA‐GFP viruses were coinjected into the mPFC of AMPKα2 flox mice. Behavioral experiments were performed 3 weeks later, and fluorescence analysis showed that the infused virus was coexpressed in the mPFC (Figure 7A). In the FUST, an decreased sniffing time for female urine (*p* = 0.0338) induced by AAV‐shRNA‐GFP treatment was ameliorated by AAV‐CaMKII‐Cre‐mCherry treatment (*p* = 0.0046; Figure 7B, *F*_(2,21)_ = 7.0200, *p* = 0.0046). Furthermore, the higher latency to food in the NSFT (*p* < 0.0010) induced by AAV‐shRNA‐GFP treatment was also blocked by AAV‐CaMKII‐Cre‐mCherry treatment (*p* = 0.0484; *F*_(2,21)_ = 10.4900, *p* < 0.0010); however, we found no significant differences in food intake (Figure 7C, treatment: *F*_(2,21)_ = 1.3070, *p* = 0.2918; timepoint: *F*_(2,42)_ = 132.2000, *p* < 0.0010; treatment × timepoint, *F*_(4,42)_ = 0.0957, *p* = 0.9833). Moreover, increased immobility time (*p* = 0.0297) in the AAV‐shRNA‐GFP treatment mice was also blocked by AAV‐CaMKII‐Cre‐mCherry treatment (Figure 7D, *p* = 0.0011; *F*_(2,21)_ = 9.1390, *p* = 0.0014). Moreover, we found no changes in the locomotor activity of these mice, demonstrating the specificity of the behavioral response (Figure 7E, treatment: *F*_(2,21)_ = 0.2660, *p* = 0.7690; timepoint: *F*_(14,294)_ = 20.0500, *p* < 0.0010; treatment × timepoint, *F*_(28,294)_ = 1.3170, *p* = 0.1361; total distance: Kruskal–Wallis test, *p* = 0.9489). Finally, we found that the mRNA of PPM1F robustly decreased with AAV‐shRNA‐GFP virus treatment (*p* = 0.0068; *p* = 0.0024, *F*_(2,15)_ = 9.8990, *p* = 0.0018), and the mRNA of AMPK α2 exon2 was also suppressed by AAV‐CaMKII‐Cre‐mCherry treatment (*p* = 0.0015; *p* = 0.0023, *F*_(2,15)_ = 12.0900, *p* < 0.0010; Figure 7F). However, the mRNA of p300 was significantly reduced by PPM1F knockdown, which was reversed by AMPK knockout (Figure 7F, *p* = 0.0072; *p* = 0.0178, *F*_(2,15)_ = 7.6150, *p* = 0.0052). Overall, inhibiting AMPK activity was found to impede the depression‐related phenotypes induced by PPM1F knockdown in the mPFC.

### 3.9 Regulation of depression‐related behaviors and stress sensitivity by inhibiting the acetylase activity of p300

Acetylase activity is the key functional component of p300;^16^, ^23^ therefore, we further explored whether the acetylase activity of p300 could be involved in inducing depression‐related behaviors. C646,^38^ a competitive p300 histone acetyltransferase, was applied to the mPFC of WT mice once daily for five days, and then the depression‐related behaviors were evaluated (Figure S9A). We observed no significant changes in sniffing time for either water (*p* > 0.9999) or female urine (*p* > 0.9999; Figure S9B, treatment: *F*_(1,23)_ = 0.1270, *p* = 0.7248; sniffing object: *F*_(1,23)_ = 287.5000, *p* < 0.0010; treatment × sniffing object, *F*_(1,23)_ = 0.2554, *p* = 0.6181), or in sucrose preference (Figure S9C, *t*_(23)_ = 0.6829, *p* = 0.5015) in the C646 and control treated mice. Moreover, these mice were exposed to subthreshold CUS conditions,^28^ and we measured their behavioral response to the stress. The results showed that the sniffing times for both water (*p* > 0.9999) and female urine (*p* > 0.9999; Figure S9D, treatment: *F*_(1,23)_ = 0.1584, *p* = 0.6943; sniffing object: *F*_(1,23)_ = 183.4000, *p* < 0.0010; treatment × sniffing object, *F*_(1,23)_ = 0.0303, *p* = 0.8633), as well as sucrose preference (Figure S9E, Mann–Whitney U test, *p* = 0.8938) were similar between the C646 and control treated mice. Immobility time in the FST (Figure S9F, Mann–Whitney U test, *p* = 0.2254) and locomotor activity were comparable between both groups (Figure S9G, treatment: *F*_(1,23)_ = 1.5540, *p* = 0.2252; timepoint: *F*_(14,322)_ = 23.3800, *p* < 0.0010; treatment × timepoint, *F*_(14,322)_ = 0.5169, *p* = 0.9230; total distance: *t*_(23)_ = 1.2460, *p* = 0.2252). The intra‐mPFC bilateral injection sites planted with canulations were correctly validated (Figure S9H).

### 3.10 Inhibiting the acetylase activity of p300 abolished the beneficial effects of PPM1F elevation in CUS‐exposed mice

**FIGURE 8:**
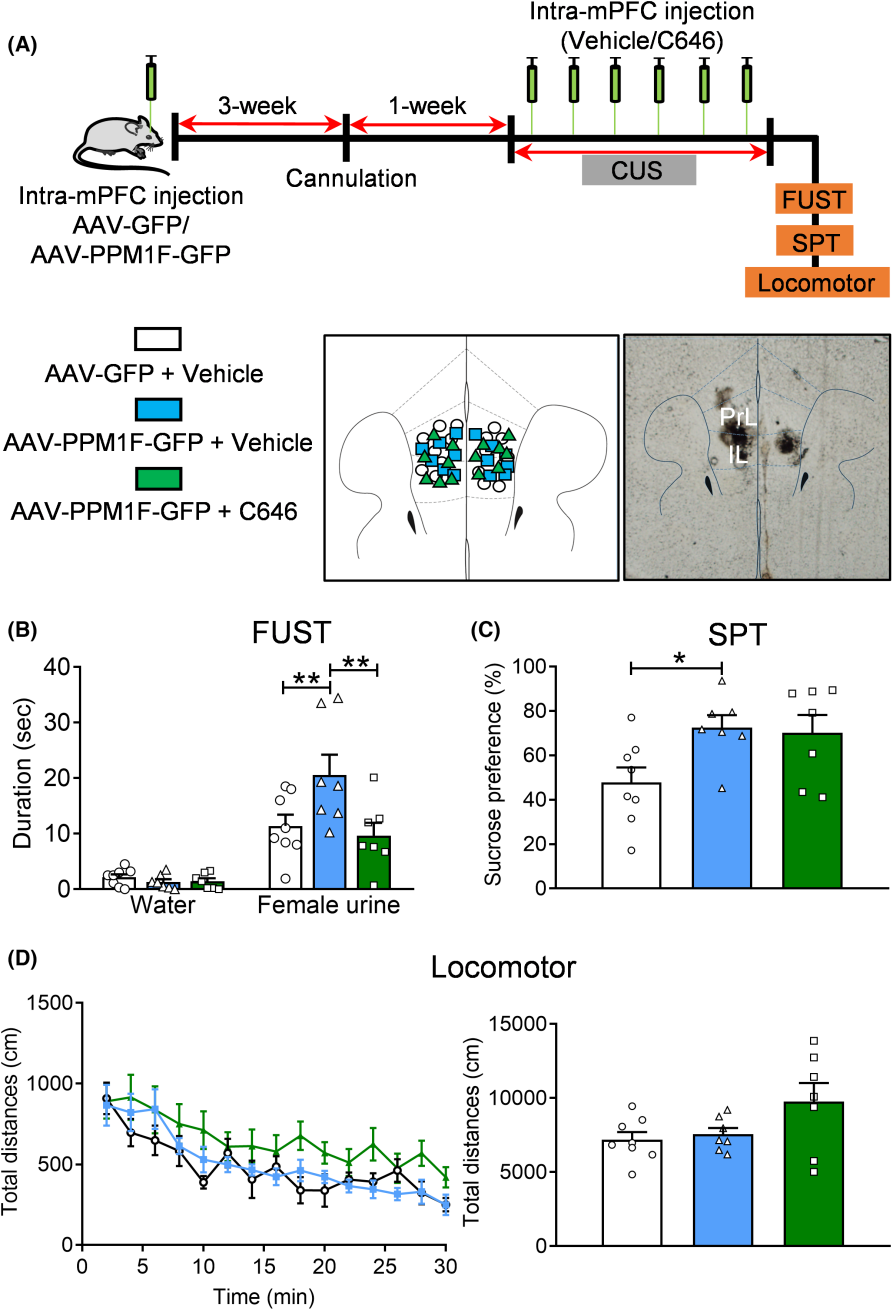
C646 blocks the antidepressant activity of PPM1F overexpression in the mPFC. (A) Upper panel: Schematic diagram of the experimental timeline. Lower panel, histological verification, and hits map of the intra‐mPFC injection sites. (B) Female urine sniffing test. (C) Sucrose preference test. (D) Locomotor activity. AAV‐GFP + Vehicle group: *n* = 8, AAV‐PPM1F‐GFP + Vehicle group: *n* = 7; AAV‐PPM1F‐GFP + C646 group: *n* = 7. *p < 0.05, **p < 0.01, compared to the control group.

To examine whether the acetylase activity of p300 mediates the antidepressant activity induced by PPM1F overexpression in the mPFC, AAV‐PPM1F‐GFP or control virus AAV‐GFP was injected into the mPFC, and these mice were then canulated 3 weeks later. Then, 1 week later, C646 was infused into the mPFC once every 2 days for 14 days. These mice were exposed to CUS, and their depression‐related behaviors were evaluated (Figure 8A). The results indicated that increased sniffing time for female urine (*p* = 0.0050) in the FUST produced by AAV‐PPM1F‐GFP treatment was reversed by C646 treatment (*p* = 0.0012; Figure 8B, treatment: *F*_(2,19)_ = 4.4010, *p* = 0.0269; sniffing object: *F*_(1,19)_ = 55.8100, *p* < 0.0010; treatment × sniffing object, *F*_(2,19)_ = 4.5020, *p* = 0.0251). However, increased sucrose preference (*p* = 0.0469) induced by AAV‐PPM1F‐GFP treatment was not affected by the C646 infusion (*p* = 0.9667; Figure 8C, *F*_(2,19)_ = 4.1040, *p* = 0.0330). Finally, we observed no changes in the locomotor activity of these mice to consolidate the specificity of the behavioral response (Figure 8D, treatment: *F*_(2,19)_ = 2.9460, *p* = 0.0768; timepoint: *F*_(14,266)_ = 17.2300, *p* < 0.0010; treatment × timepoint, *F*(_28,266)_ = 0.9082, *p* = 0.6031; total distance: *F*_(2,19)_ = 2.9460, *p* = 0.0768).

## 4 DISCUSSION

In the present study, we found that the expression of PPM1F was enclosed in the excitatory pyramidal neurons in the mPFC, and the abnormal expression of PPM1F played a pivotal role in the process of depression. shRNA‐mediated genetic knockdown of PPM1F in the mPFC produced depression‐related behaviors, while overexpression of PPM1F alleviated these behavioral responses in CUS‐exposed mice. Moreover, PPM1F exerted a regulatory function by affecting the excitability of pyramidal neurons in the mPFC and controlled the expression of p300 via the hyperphosphorylation of AMPK.

Determination of pathological genes is a direct and critical method of identifying the path for understanding depression or other mental disorders.^39^ Our previous studies revealed that significant upregulation of PPM1F in the hippocampus is related to the behavioral and pathological states of depression and anxiety.^12^, ^13^ However, in the current study, it was interesting to find that the expression levels of PPM1F in depressed mice decreased in the mPFC, another morbigenous region underlying the pathophysiology of depression,^2^, ^6^, ^40^ and this result was consistent with the another research that the reduced expression levels of PPM1F in mPFC was observed in the mice subjected to acute immobilization stress.^14^ Furthermore, downregulation of PPM1F in the mPFC increased depressive behaviors, validating the causal relationship between PPM1F and depression. Another critical founding was that the only depressed male mice displayed the reduced expression levels of PPM1F in mPFC, demonstrating the gender difference phenomenon, which was in line with the reported results that PPM1F protein levels and colocalization with CAMKIIG were altered in mPFC of only male mice after immobilization stress.^14^ However, inconsistent with our previous studies, the levels of PPM1F in the hippocampus was increased of depressed mice of both sexes, and was elevated only in female anxious mice.^12^, ^13^ The above results indicated the mixed gender and brain region specific characteristics of dysfunctional levels of PPM1F in depression.^41^ In addition, there were also slightly sex differences in the depression‐ or anxiety‐related behaviors after PPM1F knockdown in mPFC, with more prominent phenotypes in male than female, the anxious behaviors was obvious in females than in males, while the anxiolytic activity was apparent in males than in females. There are some possible explanations for these characteristics, such as the existing sex differences in corticosterone levels following CUS,^42^ and corticosterone was reported to regulate the expression of PPM1F.^14^ Moreover, the estrogen signaling system has diverse effects in the morphology and activity of mPFC neurons.^43^ The regulated molecular pathways and epigenetic modifications by PPM1F also vary between the sexes.^13^

Abnormal neuronal excitability has been well recognized as one magnified characteristic of the etiology and pathogenesis of depression.^2^, ^44^ Notably, the exact role of the neuronal excitability of the mPFC pyramidal neurons in depression was ambiguous, because some reports from our group and another group indicate these neurons show a hypoactive state in depression,^24^, ^45^ while the hyperexcitable state of these neurons in depression has also been recorded.^46^, ^47^ These contradictory results may be related to the existing fact for region‐ or projection‐specific characteristics of these neurons. Nevertheless, electrical or optogenetic activation of the mPFC has been demonstrated to exert an antidepressant effect, with reduced depressive behavioral in the FST and alleviation of chronic stress‐induced depressive phenotypes.^48^, ^49^, ^50^ In the present study, lower neuronal excitability in the pyramidal neurons in the mPFC was also observed in the excitatory neural‐specific PPM1F knockdown mice, while restoring this hypoactive condition alleviated the depressive phenotypes induced by PPM1F knockdown. Interestingly, unlike the none altered levels of hippocampal kcnn1 and kcnf1 after PPM1F overexpression,^12^ we observed that the expression levels of kcnn1 and kcnf1 were suppressed after PPM1F knockdown, which may mediate the hyperexcitability induced by PPM1F knockdown.^51^ Therefore, our results indicate that the hypoactive condition of pyramidal neurons is a key factor in the pathological process of depression, but the exact roles of kcnn1 and kcnf1 in affecting the neural excitability still need further determination.

AMPK is a serine/threonine kinase that functions as a key energy sensor in a wide variety of tissues and as a neuroprotective protein in the brain.^52^ Some research conducted by our group and others has reported that AMPK can be dephosphorylated by PPM1F or other protein phosphatase 2C family proteins.^12^, ^53^ Some studies have shown that rosiglitazone or metformin can exert an antidepressant effect in unpredictable stress‐induced depressive mouse models by activating the AMPK signaling pathway,^54^, ^55^ and directly igniting AMPK itself by activating the 5‐aminoimidazole‐4‐carboxamide‐1‐β‐d‐ribonucleotide (AICAR) also produces antidepressant effects.^56^ However, a systematic understanding of AMPK dysfunction in depression is currently lacking in the literature. We found that PPM1F knockdown in the mPFC can result in the activation of AMPK with an elevated level of p‐T172, which is consistent with the inhibitory effect of PPM1F overexpression on AMPK activation in the hippocampus.^12^ These results suggest that AMPK may be the underlying signaling protein of PPM1F in depression. Further investigation revealed that specific knockout of AMPKα2, in which subunit the phosphorylation site of threonine 172 is localized, produced antidepressive behaviors. Moreover, we also demonstrated that AMPKα2 mediates the depressive behaviors induced by PPM1F knockdown in the mPFC. To our knowledge, our study is the first to demonstrate the function of AMPK in the emergence of depression, and it also displayed as a precious participant in the role of PPM1F in depression. The unexpected and notable results in this study were the opposite modulation of PPM1F expression in the mPFC of CUS treated mice compared to the hippocampal expression levels in our previous reports,^12^ with the discrepant role in affecting depression‐related behaviors and underlying mechanism. In detail, CUS reduced the expression levels of PPM1F in mPFC, while elevated its levels in the hippocampus, knockdown of PPM1F in mPFC induced depression‐related behaviors, but produced antidepressant activity in hippocampus, accompanied with the different and individual effect on the neural excitability and signaling pathways. Nevertheless, the concrete reasons remain unclear and intricate, the diverse neural circuit connections and subregion‐specific or rostral‐caudal axis‐specific reactivity to stress may account for this divergence,^2^, ^57^, ^58^, ^59^, ^60^ which needs further exploration in the future.

We placed specific focus on acetylase p300 based on our previous research, which showed that p300 could mediate the antidepressant effect of leptin by regulating the expression of BDNF through histone H3 acetylation.^23^ In addition to its present role in serving as the presence of histone acetyltransferase activity, which endows p300 with the capacity to modulate structure‐sensitive chromatin with histone acetylation modifications, p300, as a transcriptional coactivator protein, plays a central role in coordinating and regulating many signal‐dependent events with the transcription process and other multiple ways.^16^ For example, it acts as a protein bridge, thereby connecting diverse transcription factors to transcription sites and also provides a protein scaffold for the formation of a multicomponent transcriptional regulatory complex. However, the exact role and upstream molecular modifier(s) of p300 remain unclear. Thus, AMPK can directly phosphorylate and downregulate p300 mediated acetylation and transcription functions,^34^, ^61^ which suggests that AMPK may be a potential modulator of p300 activity. Therefore, we raised the hypnosis that depression was developed from the downregulation of PPM1F, which enables the activation of AMPK, leading to the blocking effect of the p300‐associated transcriptional promoter. Meanwhile, our results validated the hypothesis that decreased p300 expression levels occurred after PPM1F knockdown, which was abolished by AMPK knockout. However, an unexpected finding was that the infusion of C646, which is a small‐molecule specific inhibitor of p300, to the mPFC, could not induce depression‐related behaviors in either the basal or subthreshold CUS conditions, but partially blocked the antidepressive effect of PPM1F overexpression. These features have some potential explanations, the first one we must remain is that the functional dimorphic characters of the subregions of mPFC, prelimbic (PrL) and infralimbic (IL) of the mPFC;^24^, ^50^, ^62^, ^63^ therefore, the infusion strategy might be improved to promote the location accuracy. Another possible explanation is that complicated types of neurons, pyramidal neurons, inhibitory GABAergic interneurons and excitatory interneurons coexist in this region; thus, the infused C646 may have a comprehensive effect. Furthermore, p300 is a nuclear phosphoprotein, and apart from AMPK, many kinases have been implicated in the phosphorylation process.^16^, ^64^ The exact role of p300 in depression also awaits further elucidation using brain regions or neural‐specific manipulative strategies in the future.

In summary, this study's results indicate that PPM1F‐AMPK‐p300 activity in the mPFC neurons is a key regulator of depression‐related behaviors, and our data suggest that abnormal PPM1F activity, impaired neuronal excitability in the pyramidal neurons and enhanced neuroinflammation state may contribute to the development of depression. However, how chronic stress causes a change in PPM1F expression in the mPFC, the neuronal populations and targeted genes of p300 mediating regulation of the effects of PPM1F on depression require further exploration.

## AUTHOR CONTRIBUTIONS

Jing Liu, Fantao Meng, and Wentao Wang: Methodology, Investigation, Funding acquisition. Min Wu and Yu Zhang: Methodology, Project administration. Minghu Cui and Changyun Qiu, Fengai Hu, Di Zhao, Dan Wang and Cuilan Liu Methodology, Resources software. Dunjiang Liu, Zhicheng Xu, and Yameng Wang: Methodology and Software. Chen Li and Wei Li: Writing—original draft, data curation, funding acquisition, supervision, project administration.

## FUNDING INFORMATION

This work was supported by the Shandong Provincial Natural Science Foundation (ZR2022QH172 to JL, ZR2022YQ65 and ZR2021MH073 to CL; ZR2019PH109 to WTW), the National Natural Science Foundation of China (82171521 to CL), the Special Funds of Taishan Scholars Project of Shandong Province (NO. tsqn202211368 to CL), and the Projects of Medical and Health Technology Development Program in Shandong Province, China (202003090720; 202003070728).

## CONFLICT OF INTEREST STATEMENT

The authors declare that they have no conflicts of interest.

## Supporting information


Figure S1

Figure S2

Figure S3

Figure S4

Figure S5

Figure S6

Figure S7

Figure S8

Figure S9

Table S1

## Data Availability

The datasets supporting the conclusions of this study are included within the article. The data that support the findings of this study are available from the corresponding author upon reasonable request.
