# Supplementary figures and images for: Medial prefrontal cortical PPM1F alters depression‐related behaviors by modifying p300 activity via the AMPK signaling pathway
Source: CNS Neurosci Ther. 2023 Jun 12;29(11):3624–43. doi: 10.1111/cns.14293 (PMC10580341; doi:10.1111/cns.14293)

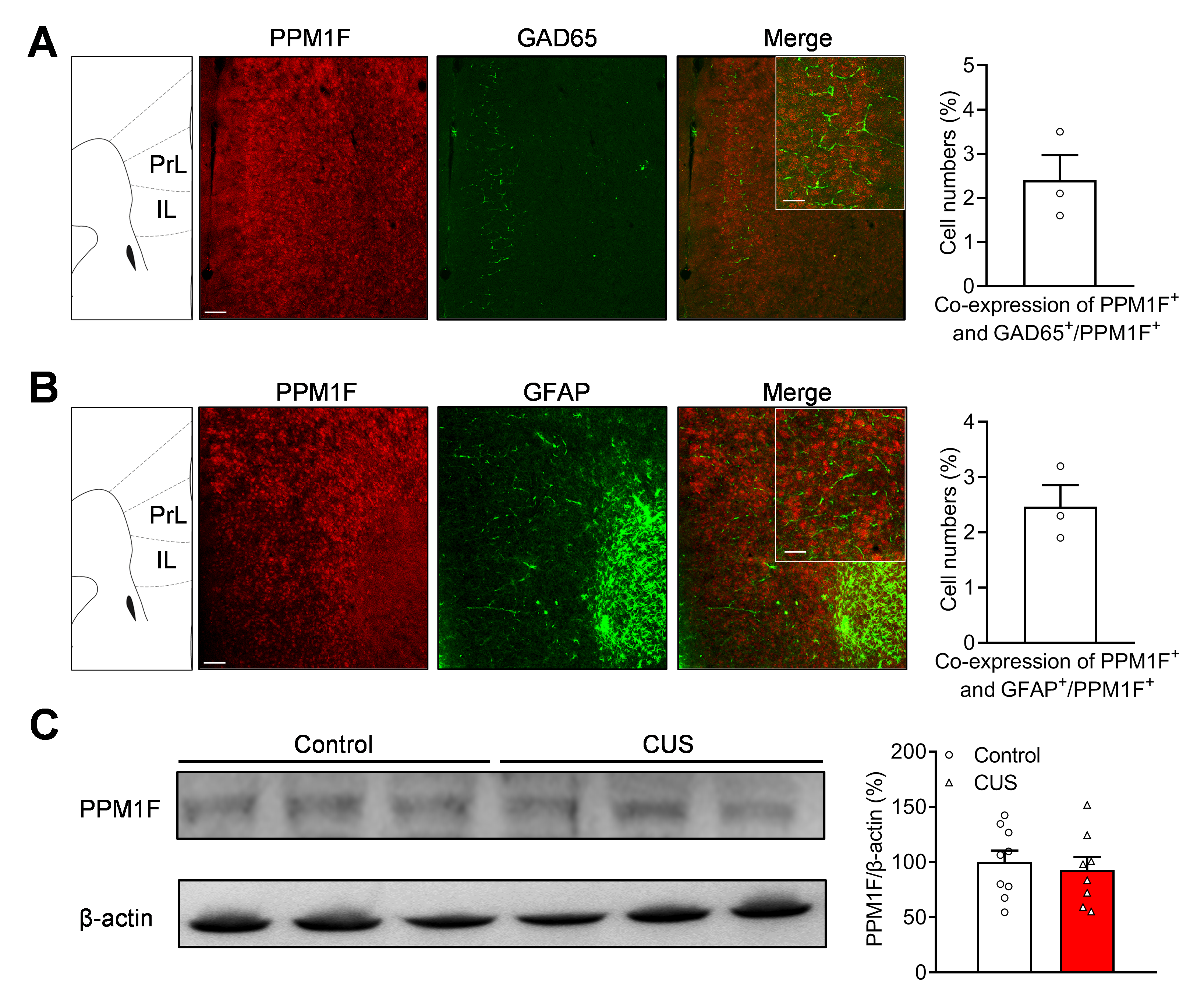

Supplement: Supplementary file 1 — Figure S1 [file CNS-29-3624-s008.tif]

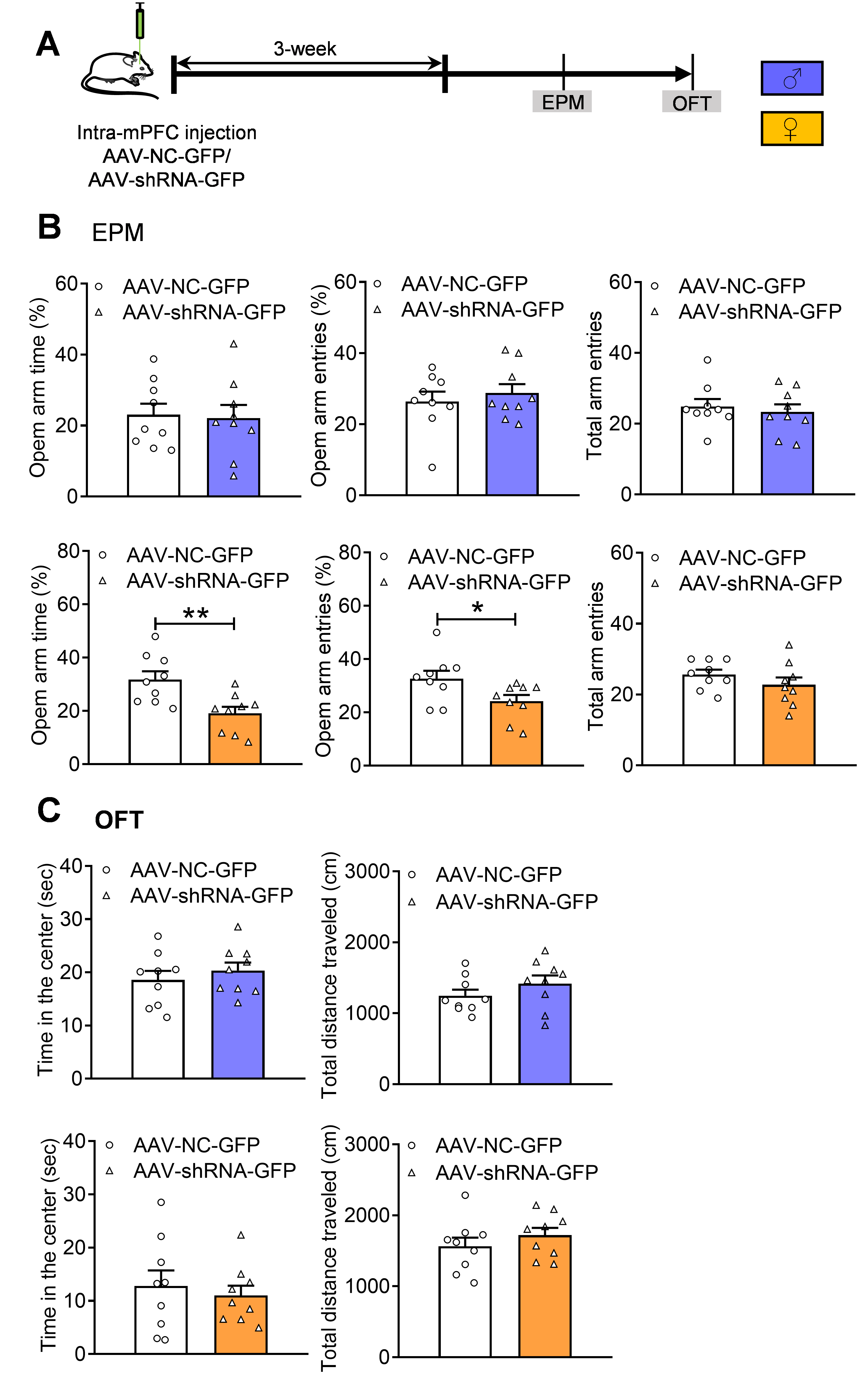

Supplement: Supplementary file 2 — Figure S2 [file CNS-29-3624-s006.tif]

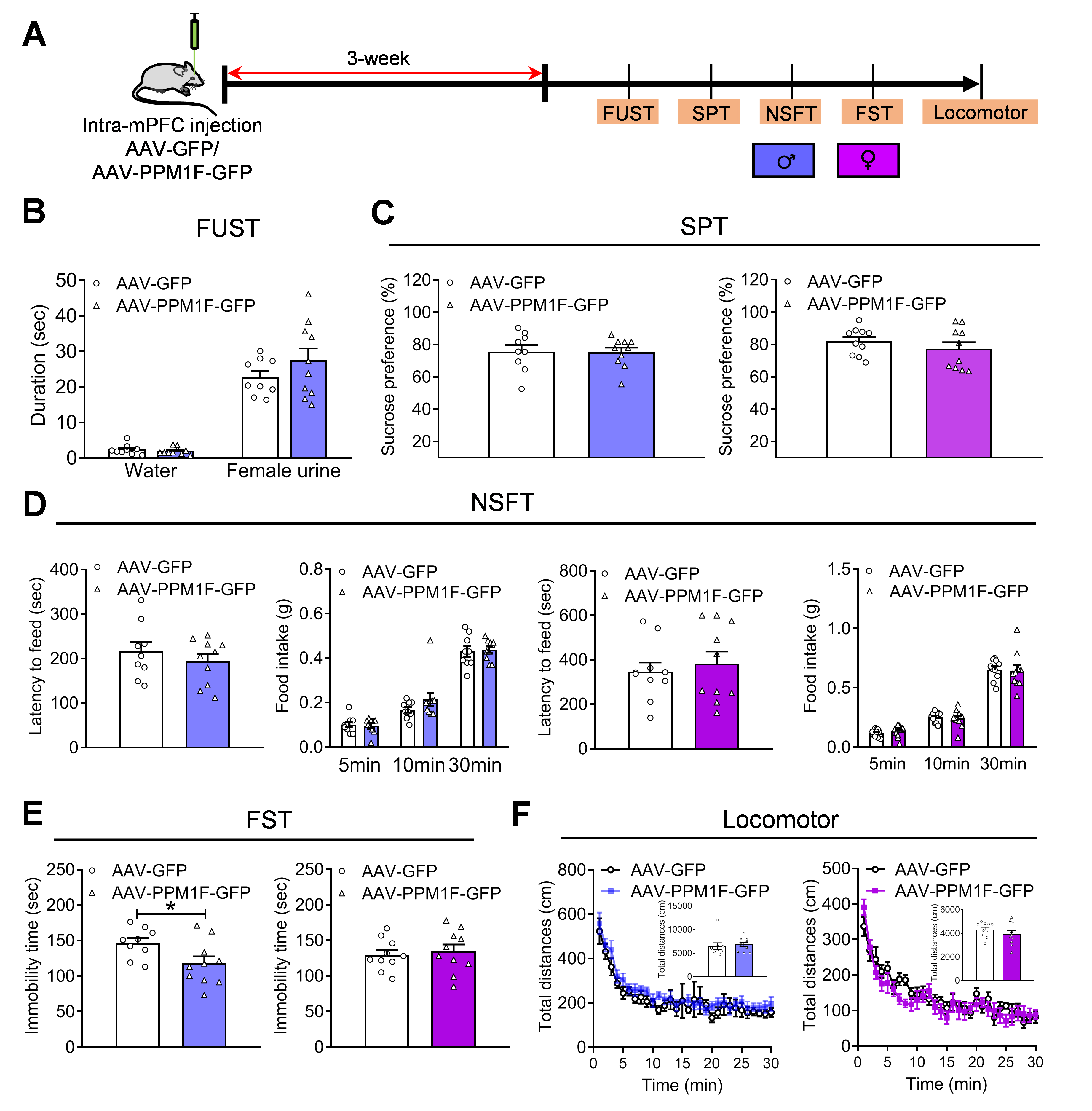

Supplement: Supplementary file 3 — Figure S3 [file CNS-29-3624-s003.tif]

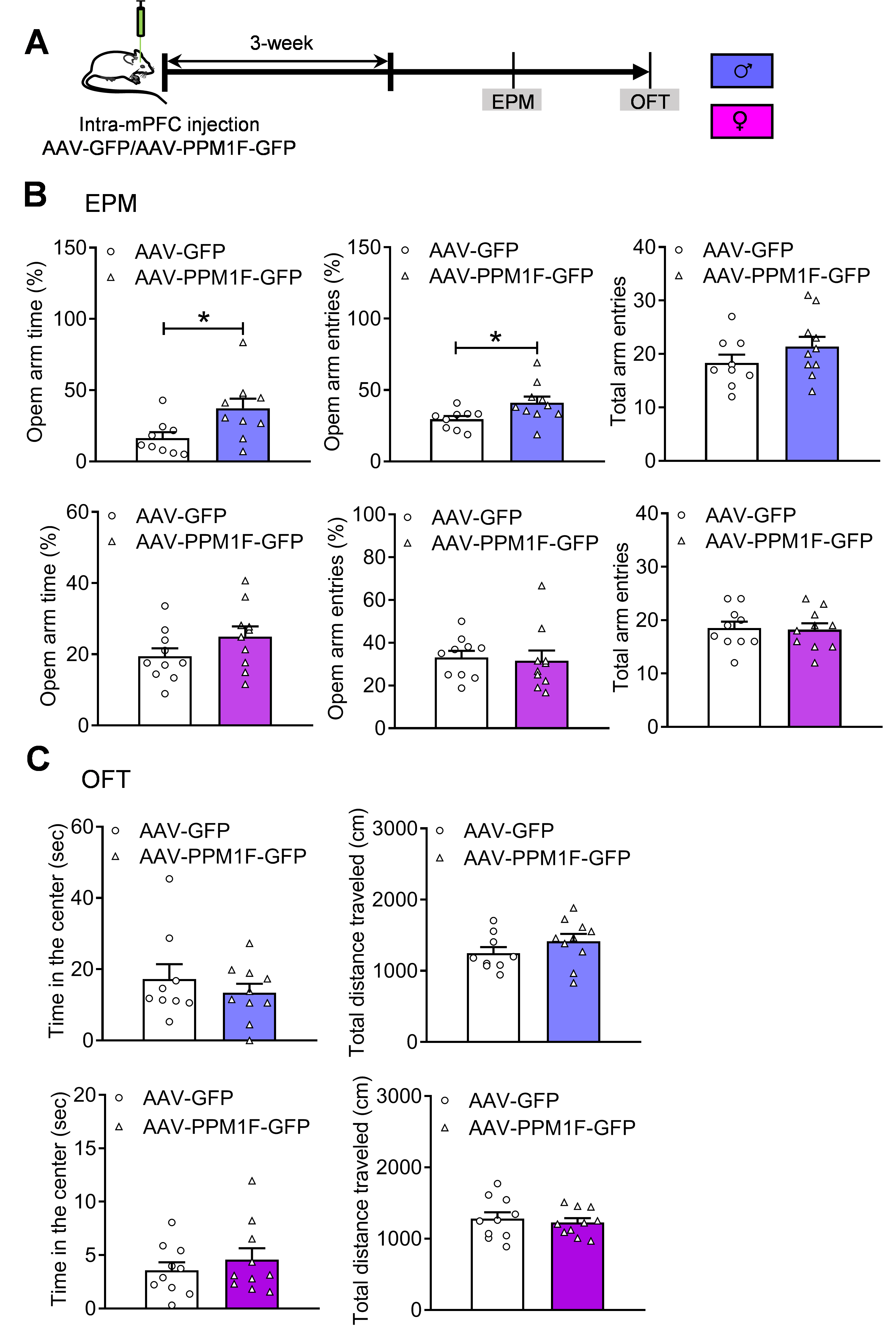

Supplement: Supplementary file 4 — Figure S4 [file CNS-29-3624-s002.tif]

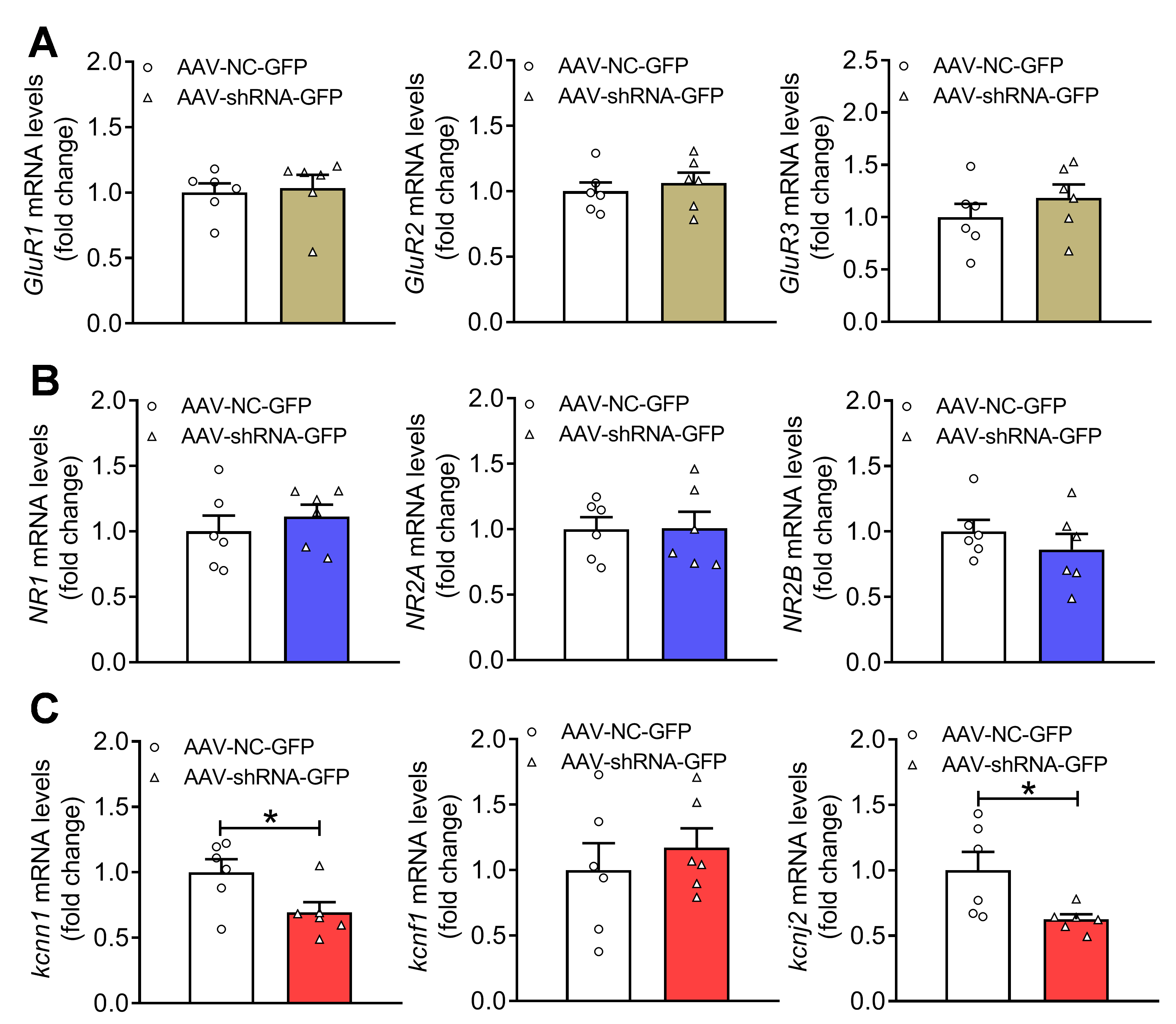

Supplement: Supplementary file 5 — Figure S5 [file CNS-29-3624-s010.tif]

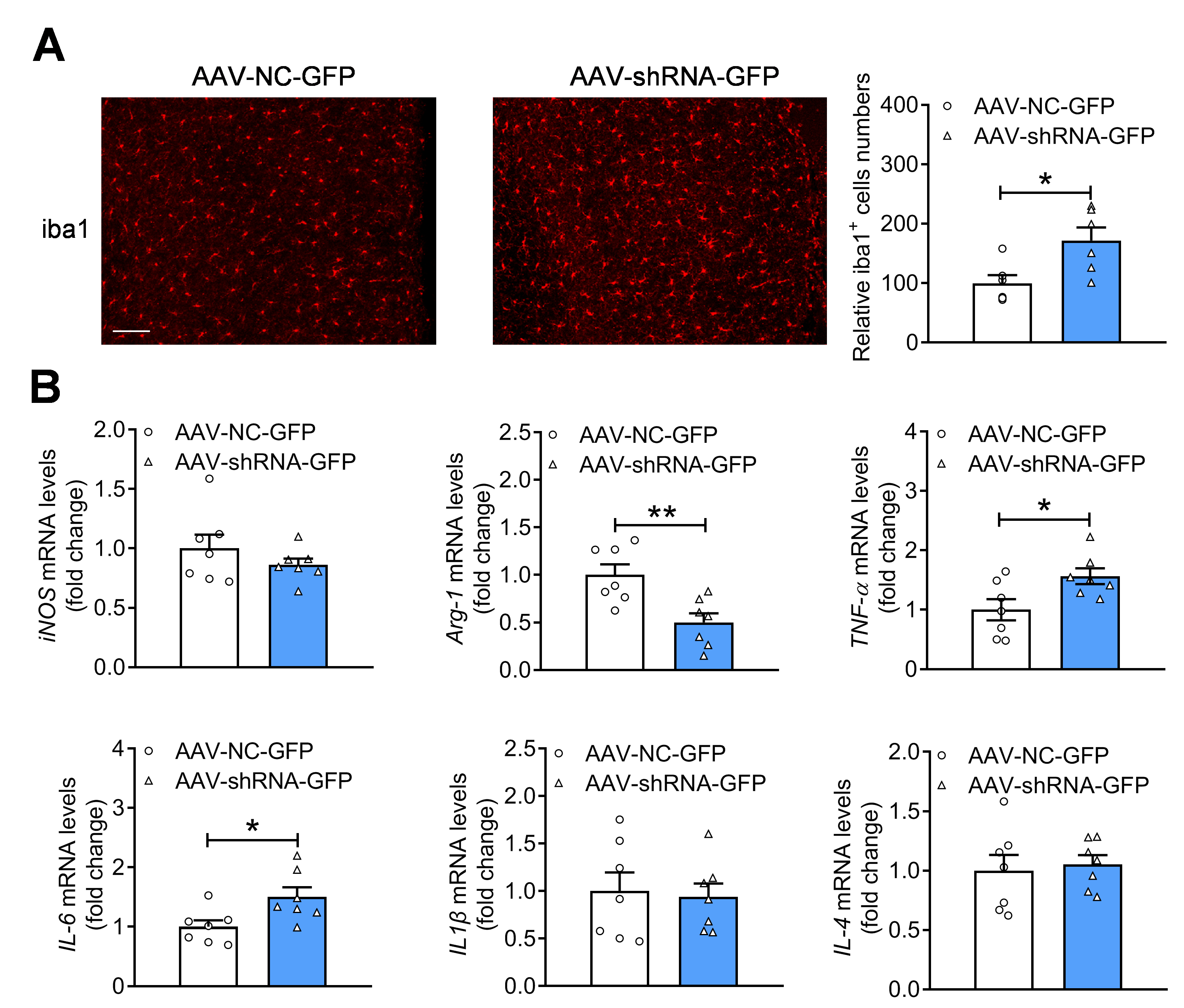

Supplement: Supplementary file 6 — Figure S6 [file CNS-29-3624-s005.tif]

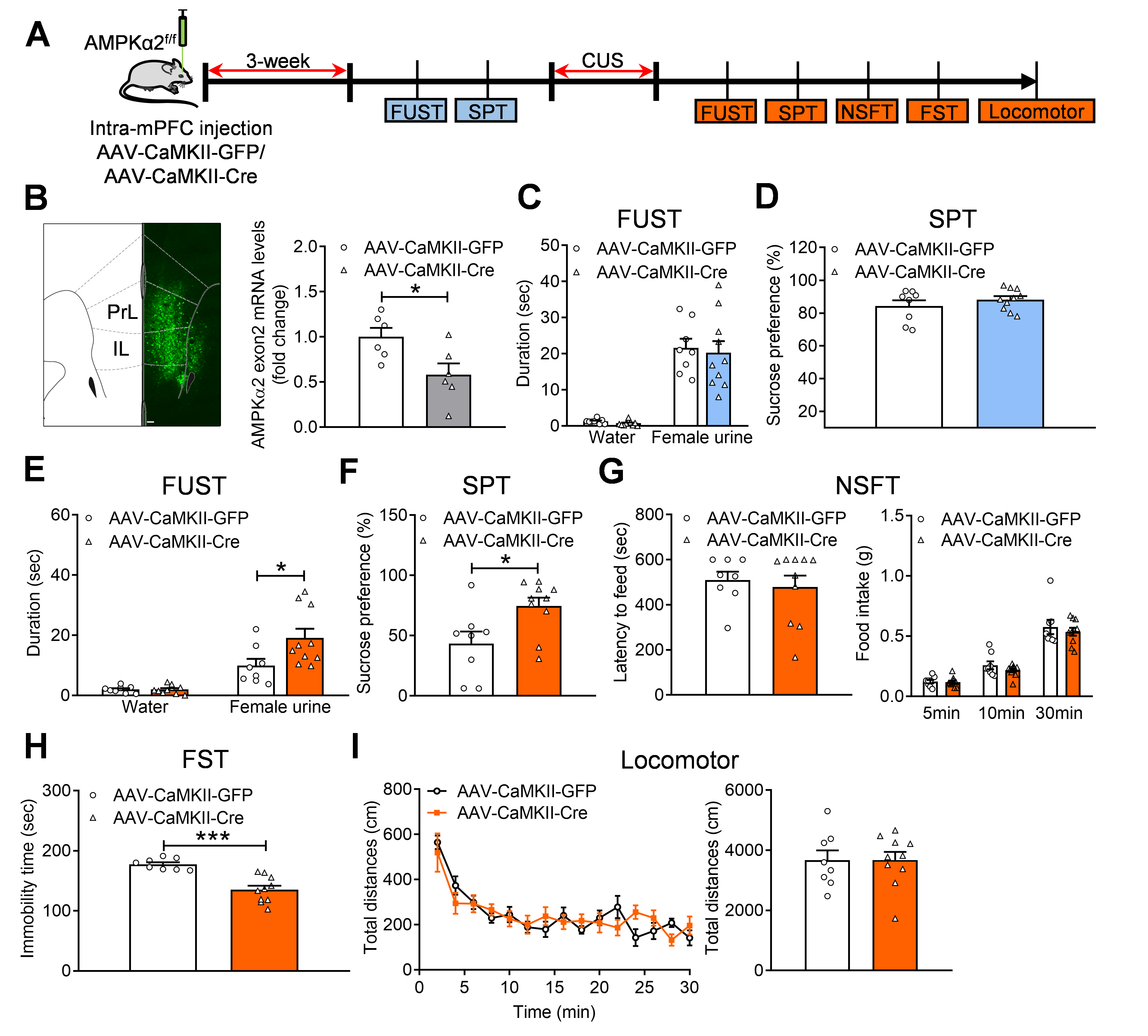

Supplement: Supplementary file 7 — Figure S7 [file CNS-29-3624-s004.tif]

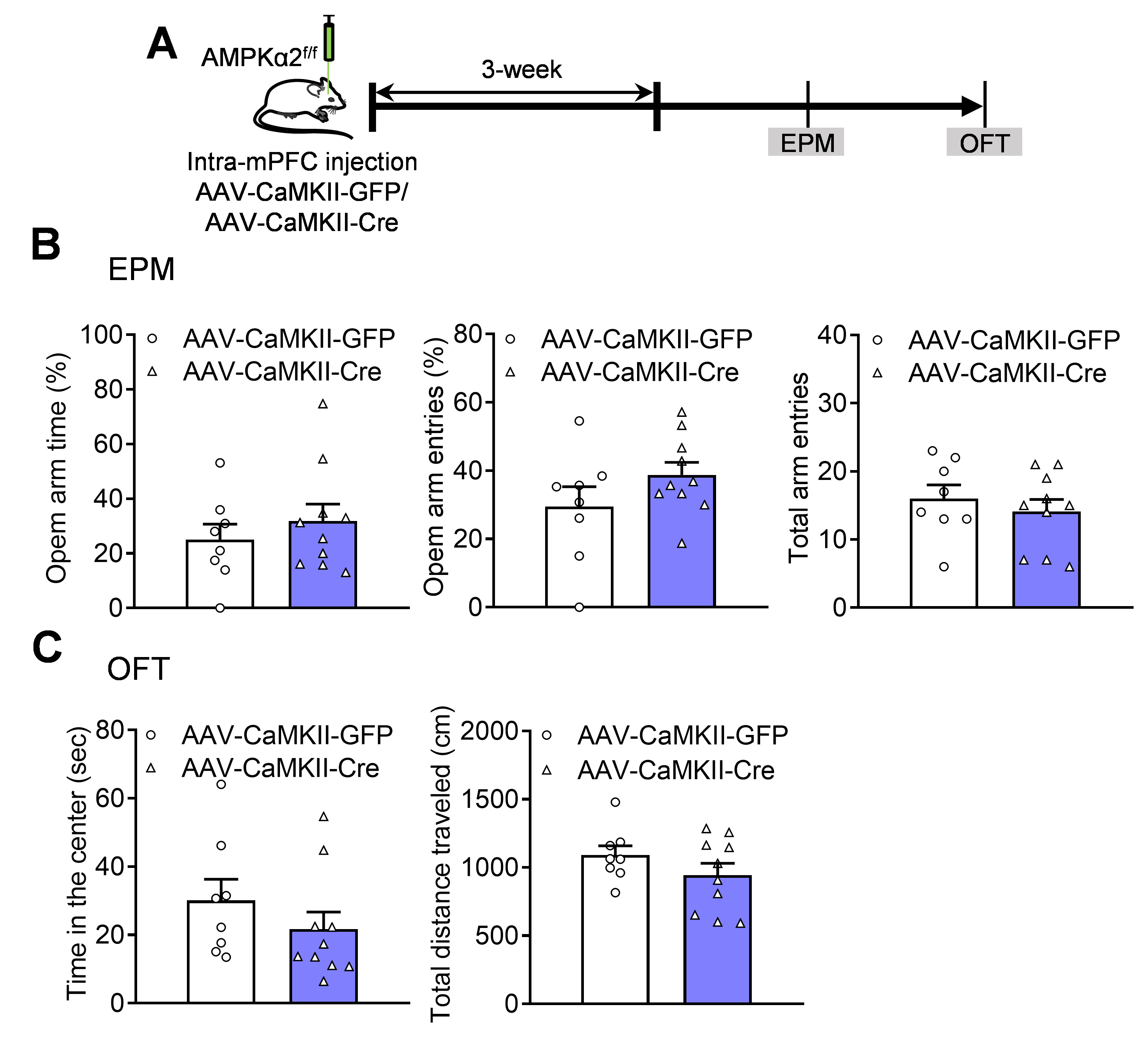

Supplement: Supplementary file 8 — Figure S8 [file CNS-29-3624-s009.tif]

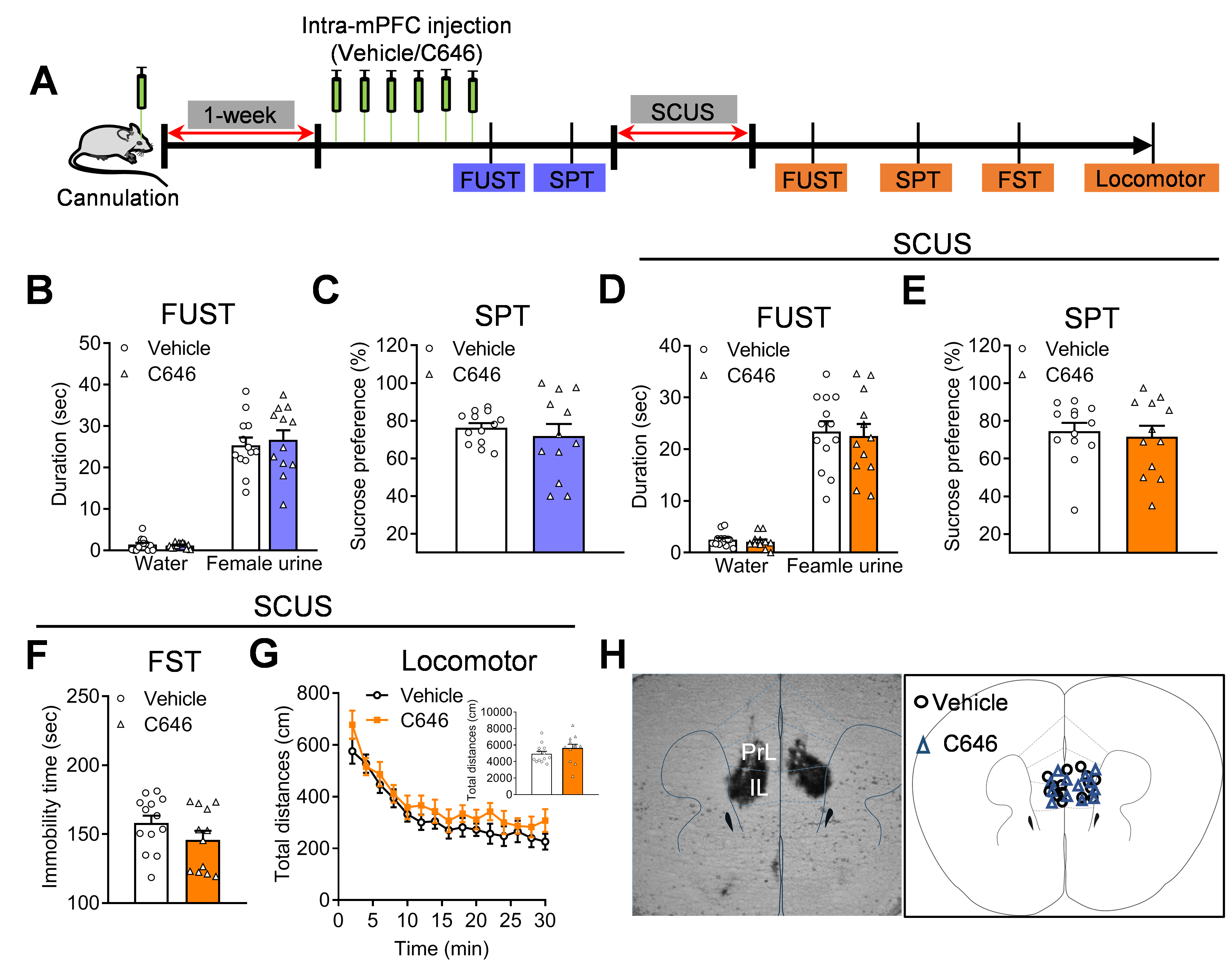

Supplement: Supplementary file 9 — Figure S9 [file CNS-29-3624-s007.tif]
