# Supplementary material for: Medial prefrontal cortical PPM1F alters depression‐related behaviors by modifying p300 activity via the AMPK signaling pathway
Source: CNS Neurosci Ther. 2023 Jun 12;29(11):3624–43. doi: 10.1111/cns.14293 (PMC10580341; doi:10.1111/cns.14293)
Supplement: Supplementary file 10 — Table S1 [file CNS-29-3624-s001.docx]

Supplemental table1: Primer sequences used for Real-time PCR analysis.

| **Gene** | **Forward primer (5'–3')** | **Reverse primer (5'–3')** |
| --- | --- | --- |
| PPM1F | AGATGGCCTGTGACAGTGAGA | AAGCCCAATGCCAGCTCAA |
| p300 | GGAGCAAGCTAATGGGGAAGTGAG | CCCCAGCATTTTTGAGAGGAAGAC |
| AMPKα2 | TCGCAGTTTAGATGTTGTTG | ATGAGGATGACGAAAGAGT |
| GluR1 | GTCCGCCCTGAGAAATCCAG | CTCGCCCTTGTCGTACCAC |
| GluR2 | TGTGTGGTGGTTCTTTACCCT | AGTAGGCATACTTCCCTTTGGAT |
| GluR3 | GTGCAGTTATACAACACCAACCA | GAGCAGAAAGCATTAGTCACAGA |
| NR1 | AGAGCCCGACCCTAAAAAGAA | CCCTCCTCCCTCTCAATAGC |
| NR2A | ACATCCACGTTCTTCCAGTTTGG | GACATGCCAGTCATAGTCCTGC |
| NR2B | GCCATGAACGAGACTGACCC | GCTTCCTGGTCCGTGTCATC |
| Kcnn1 | GCTCTTTTGCTCTGAAATGCC | CAGTCGTCGGCACCATTGTCC |
| Kcnf1 | CGTGGCAGGCGAAGACATT | CCCCCGCCAAACAGTTGAT |
| Kcnj2 | ATGGGCAGTGTGAGAACCAAC | TGGACTTTACTCTTGCCATTCC |
| β-tubulin | AGCAACATGAATGACCTGGTG | GCTTTCCCTAACCTGCTTGG |
